# The coming of age of DNA‐based catalysts for therapeutic applications

**DOI:** 10.1002/ctm2.70408

**Published:** 2025-07-14

**Authors:** Robert Hesse, Christoph G. W. Gertzen, Jessica Felice Schmuck, Justin Darvin Böcker, Piyush Pandey, Tobias Behn, Christopher Ruth, Detlev Riesner, Stephanie Kath‐Schorr, Philipp A. Lang, Holger Gohlke, Manuel Etzkorn

**Affiliations:** ^1^ Faculty of Mathematics and Natural Sciences Institute for Pharmaceutical and Medicinal Chemistry Heinrich Heine University Düsseldorf Germany; ^2^ Faculty of Mathematics and Natural Sciences Center for Structural Studies Heinrich Heine University Düsseldorf Germany; ^3^ Faculty of Mathematics and Natural Sciences Institute of Physical Biology Heinrich Heine University Düsseldorf Germany; ^4^ Department of Molecular Medicine II, Medical Faculty Heinrich Heine University Düsseldorf Germany; ^5^ Department of Chemistry Institute of Organic Chemistry University of Cologne Cologne Germany; ^6^ Institute of Bio‐ and Geosciences (IBG‐4: Bioinformatics) Forschungszentrum Jülich Jülich Germany; ^7^ Institute of Biological Information Processing (IBI‐7) Forschungszentrum Jülich Jülich Germany

**Keywords:** DNA enzymes, DNAzyme, emerging therapeutic platform technology, gene‐silencing, RNA‐cleavage

## 1 INTRODUCTION, MOTIVATION, AND EARLIER DEVELOPMENTS OF THE DNAZYME TECHNOLOGY

**FIGURE 1:**
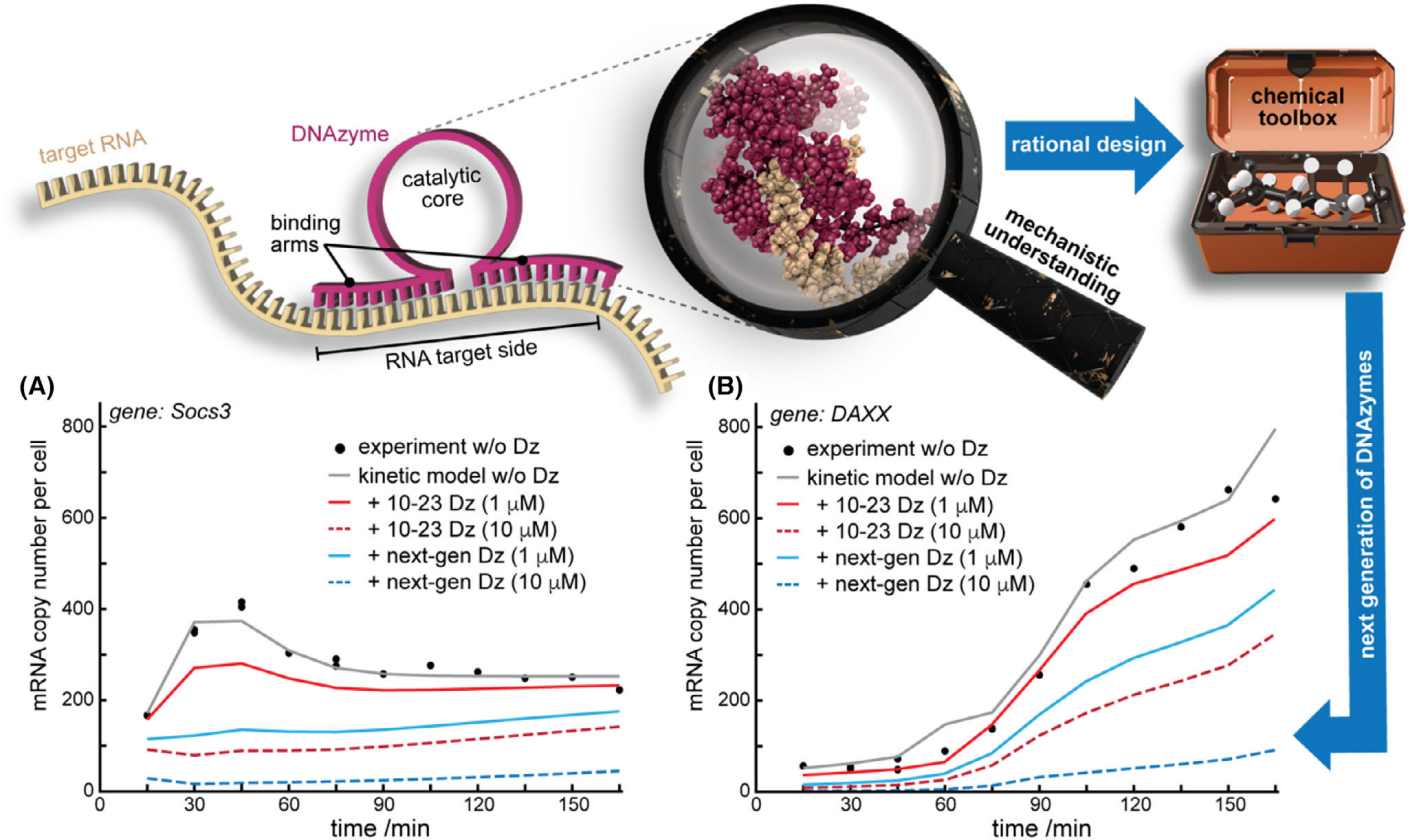
Schematic overview of the DNAzyme technology, its potential improvements via rational design, and simulated effects of improved variants. Plots in *a*+*b*) show time‐dependent cellular mRNA levels for two different mRNAs as experimentally determined in ref.^3^ (black circles). Lines represent data of the herein‐developed kinetic model to simulate the effects of DNAzyme treatment on the respective mRNA levels (see the text and Supporting information for more details). Red curves assume cleavage rates observed in vitro for a classical 10–23 Dz variant.^4^ Blue curves show the potential impact of a 6‐fold increased cleavage rate as observed for a rationally improved Dz variant.^4^ ^.^

DNA enzymes, also known as DNAzymes (Dz), are synthetic high‐precision biocatalysts that have been identified by in vitro selection three decades ago.^1^ Dz are usually short, single‐stranded DNA molecules that catalyse chemical reactions through their specific three‐dimensional structure.^2^ Due to their enormous therapeutic potential, particular interest has been invested in RNA‐cleaving Dz, such as the 8–17 Dz and 10–23 Dz^2^. In general, these DNAzymes share a modular architecture comprising a (conserved) catalytic loop sequence and adaptable substrate binding arm sequences that, following specific design guidelines, can be modified to bind virtually any given target RNA with high selectivity (Figure 1).

### 1.1 DNAzymes in clinical studies

A considerable number of Dz variants have been investigated in preclinical studies, but very few have entered clinical trials. Notably, the handful of Dz‐based drug candidates that entered clinical trials share their catalytic core sequence with the original 10–23 Dz. Three of these Dz (SB010, SB011, and SB012) have been developed by *sterna biologicals GmbH* to target different roles of the transcription factor GATA‐3. SB010 targets GATA‐3 as an important transcription factor in the allergen‐driven Th2 molecular endotype of asthma. In a double‐blind phase IIa study (NCT01743768) with 38 male patients suffering from mild allergic asthma, the orally inhaled SB010 attenuated the mean late asthmatic response by 34% and the early asthmatic response by 11% with no noteworthy differences in adverse events to the placebo group.^5^ A second phase IIa study with SB010 in moderate to severe asthma started in 2021 (Sterna Biologicals GmbH). Topically medicated SB011 exploits the role of GATA‐3 in lesional skin in patients with atopic eczema and is currently being studied in a phase IIa double‐blind clinical trial (NCT02079688). SB012, targeting GATA‐3′s role in inflammatory cytokine production in patients with ulcerative colitis,^6^ is currently studied in a phase IIa double‐blind clinical trial (NCT02129439) for intrarectal application.

The remaining Dz‐drug candidates that have entered clinical trials are Dz1 and Dz13. Dz1 targets the latent membrane protein‐1 (LMP1) mRNA of the Epstein–Barr virus (EBV) in patients with nasopharyngeal carcinoma (NPC). A clinical randomised double‐blind study on 40 NPC patients in conjunction with radiation therapy resulted in increased tumour regression compared to the control group with only radiotherapy, an undetectable level of EBV DNA copies in plasma samples, and no toxic side effects in patients.^7^ Dz13 targets the mRNA of the transcription factor c‐Jun involved in basal cell carcinoma (BCC). In a first‐in‐human phase I trial addressing the safety and tolerability of intratumorally injected Dz13 in patients with BCC, Dz13 lipoplex resulted in the reduction of tumour depth with no observed toxic side effects.^8^

Overall, the clinical data indicate that Dz treatments are safe without apparent side effects. Still, none of the candidates have entered clinical phase III level, and reasons for the apparent discontinuation of some clinical trials have not been published.

### 1.2 DNAzymes for antiviral strategies

As RNA can be regarded as the heart of virus replication, the unique antiviral potential of RNA‐cleaving Dz is imminent. Commonly used viral inhibitors often suffer from toxicity, drug resistance, and ecotoxicological consequences. Nucleic acid‐based therapies can tackle these shortcomings. Data to utilise Dz for anti‐viral therapy in the context of influenza A virus, Japanese encephalitis virus, human rhinoviruses, respiratory syncytial virus, and SARS‐CoV‐2 support the concept. The Dz13 has yielded a lower pulmonary titre 6 days post infection of the virus and a better survival rate 15 days post infection in mice infected with Influenza A virus.^9^ Likewise, during Japanese encephalitis virus infection, 3DzG reduced viral titre in mouse brain 72 h post infection and also provided increased survival until 14 days post infection.^10^ Furthermore, treatment with DNAzymes during rhinovirus infection caused a reduced virus copy number in sinonasal tissue samples.^11^ Respiratory syncytial virus (RSV) infection was successfully inhibited by DZn1133 through decreased transcription and expression of F viral gene‐fusion protein, reducing the RSV yield by about 7 log units and protecting more than 90% of RSV‐infected Hep‐2 cells from a cytopathic effect.^12^

The emergence of the COVID‐19 pandemic underscores the importance of more dynamic tools to counteract viral infections. Sufficiently bioactive DNAzymes could constitute a new toolset for virus‐specific antiviral therapeutics. Their site‐specific response also safeguards non‐targeted cellular RNA from the action of DNAzymes. Furthermore, the binding arms can be modified to bind to different segments of the viral RNA, such as shown in cell culture experiments by the FR6_1 XNAzyme targeting COVID‐19,^13^ increasing the target range to effectively suppress infection and impede resistance.

### 1.3 What is holding us back?

Despite the above outlined general potential and individual successes, the catalytic activity of 10–23 Dz variants requires the presence of divalent‐metal‐ion cofactors such as Mg^2+^; a feature that regularly leads to reduced activity under cellular conditions.^14^, ^15^, ^16^, ^17^, ^18^ Whilst notable improvements have been made (e.g., reviewed in^19^), the observed low bioactivity has been limiting the DNAzyme technology for decades. Furthermore, so far clinical strategies have focused on topical application procedures, and studies focusing on administration via the systemic route are sparse, constraining the general applicability of the Dz technology.

Comparing Dz variants with identical core sequences but different arm sequences also highlights an often‐neglected impact of the arm sequence on the Dz activity. This is regularly observed even under defined in vitro conditions using minimal RNA sequences, where the Dz activity can easily vary by one order of magnitude due to (un)favourable arm‐sequence variations. This observation as well as a reliable prediction of target‐site accessibility in the context of the full‐length RNA substrate is often inadequately incorporated in current Dz‐design procedures. Consequently, we envision that the field will considerably benefit from improvements in (i) target‐site selection algorithms and (ii) the usage of standardised experimental conditions for future studies. For the latter, we recommend using the conditions applied in, for example, ref.,^20^ for future in vitro studies as well as standardised in cellulo systems. This will be necessary to build a sufficiently large comparative database for improved pattern recognition in Dz design.

### 1.4 The coming of age of the Dz technology

The in vitro evolution of the 10–23 Dz^2^ and subsequent comprehensive systematic modifications thoroughly explored the possibilities offered by the chemical space of natural nucleotides. Therefore, non‐natural chemical modifications are a logical next step to overcome persisting limitations and further optimise the catalytic activity, metal‐ion independence, and biostability. This development is accelerated by progress in the closely related field of antisense oligonucleotides (ASO) tested in numerous clinical trials.^21^ For DNAzymes, modifications in the arms are considered to improve cellular lifetimes and annealing to the template RNA, whereas modifications in the catalytic loop aim to improve the catalytic activity, reduce Mg^2+^ dependency, and/or enable orthogonal activation.^4^, ^13^, ^22^, ^23^ Backbone and sugar modifications such as phosphorothioates (PS), FANA (2′‐deoxy‐2′‐fluoro‐arabinonucleic acid), and 2′OMe have been introduced and showed improved nuclease resistance and/or catalytic activity.^20^, ^24^, ^25^, ^26^, ^27^ However, the vast chemical space of nucleobase modifications is yet to be fully explored, which has been obstructed by an insufficient molecular understanding.

Our recent mechanistic insights into the 10–23 Dz opened new routes in the structure‐guided rational design of functionalised nucleobase residues.^4^, ^28^ Exemplary for this approach is the 6‐thio‐dG14 variant, which increases the cleavage rate by 6‐fold via supposedly reducing inactive conformations.^4^ Accordingly, a pressing question arises: are the currently developed Dz variants sufficient for a more generalised therapeutic platform?

To get a better overview of the bioactivity required to downregulate commonly occurring cellular mRNA levels, we here simulated how Dz activity relates to cellular mRNA degradation. We built a kinetic model for the time course of the total mRNA level resulting from gene transcription, RNA maturation as well as natural and Dz‐mediated RNA degradation. To do so, we recalculated the time‐dependent kinetic models of 254 genes reported before^3^ and added a term for Dz‐mediated degradation and, optionally, degradation of the Dz itself. The approach and generated data are described in detail in Supporting information. The simulations help to understand how an increased cleavage rate, such as observed for the 6‐thio‐G14 variant in vitro,^4^ could propagate to the cellular level of the targeted mRNA (Figure 1a,b; red vs. blue curves). Under the applied conditions, the improved variant, unlike the non‐modified Dz, could flatten the Socs3 mRNA levels already at a 10‐fold lower dosage (Figure 1a). For the Daxx system, the effects are even more critical, suggesting that therapeutic effects involving a particular mRNA expression behaviour will strongly depend on the Dz cleavage rate (Figure 1b, also see Supporting information for data on all other mRNA transcripts).

In general, new variants with further increased bioactivity and biostability will reduce the required Dz concentrations, which should lower immune response and facilitate administration. Due to the Dz's comparatively small molecular size and high stability, most formulations developed for ASO, siRNA, and/or mRNA delivery should be well applicable also for Dz treatment. Still, suitable formulations capable of delivering the required Dz concentrations and cell type‐specific reduced activities may restrict certain applications and will require further optimisation.

## 2 CONCLUSIONS

In summary, persisting limitations of the Dz technology for therapeutic applications are on the verge of being resolved. The recent progress is largely based on the usage of non‐natural modifications for (i) improved Dz arm compositions enhancing cellular stability and RNA interactions, (ii) advanced experimental screening of (non‐natural) catalytic core sequences, and (iii) structure/dynamics‐guided rational design strategies. Due to its favourable performance under physiologically relevant conditions, we here focused on the 10–23 types of Dz. However, progress on other RNA‐cleaving Dz types (e.g.,^24^, ^29^, ^30^, ^31^, ^32^) may add additional features to the field. Bringing together the progress in different areas, it can be anticipated that new Dz variants with further increased bioactivity will be iteratively developed. Our *Gedanken experiment* suggests that increased cleavage rates will be particularly important for specific mRNA transcription behaviours (Figure 1b), and that transfer of the observed (in vitro) catalytic efficiency of the current Dz variants to the target cells should be sufficient for a wide range of therapeutic applications. The currently developed new generation of Dz shows great promise in realising this task and, in combination with appropriate selection algorithms and delivery systems, should offer a robust modular platform capable of downregulating a broad spectrum of target RNAs. Just in time for its 30th birthday, the Dz technology is hence coming of age, paving the way for innovative therapeutic approaches in areas as diverse as animal health, plant protection, and human viral/bacterial infections, cancer, neurodegeneration, personalised medicine, and rare diseases.

## AUTHOR CONTRIBUTIONS

R.H., C.G.W.G., and H.G. developed the presented kinetic model/simulation of the Dz‐mediated reduction of mRNA levels. All authors participated in evaluation of previous literature and/or clinical data as well as conceptualised and wrote the article.

## CONFLICT OF INTEREST STATEMENT

The authors declare no conflicts of interest.

## ETHICS STATEMENT

Not applicable.

## Supporting information



Supporting information
