# Supplementary material for: The coming of age of DNA‐based catalysts for therapeutic applications
Source: Clin Transl Med. 2025 Jul 14;15(7):e70408. doi: 10.1002/ctm2.70408 (PMC12260117; doi:10.1002/ctm2.70408)
Supplement: Supplementary file 1 — Supporting information [file CTM2-15-e70408-s001.pdf]

Suppplementary Information

The coming of age of DNA-based catalysts for therapeutic applications

Robert Hesse[1], Christoph W.G. Gertzen[1,2], Jessica F. Schmuck[3], Justin Böcker[3], Piyush Pandey[4], Tobias Behn[5], Christopher Ruth[3], Detlev Riesner[3], Stephanie Kath-Schorr[5]*, Philipp A. Lang[4]*, Holger Gohlke[1,6]*, Manuel Etzkorn[3,7,]*

Affiliations

[1]Heinrich Heine University, Faculty of Mathematics and Natural Sciences, Institute for Pharmaceutical and Medicinal Chemistry, Düsseldorf, Germany

[2]Heinrich Heine University, Faculty of Mathematics and Natural Sciences, Center for Structural Studies, Düsseldorf, Germany

[3]Heinrich Heine University, Faculty of Mathematics and Natural Sciences, Institute of Physical Biology, Düsseldorf, Germany

[4]Department of Molecular Medicine II, Medical Faculty Heinrich Heine University, Germany

[5]Institute of Organic Chemistry, Department of Chemistry, University of Cologne, Germany

[6]Institute of Bio- and Geosciences (IBG-4: Bioinformatics), Forschungszentrum Jülich, Jülich, Germany

[7]Institute of Biological Information Processing (IBI-7), Forschungszentrum Jülich, Jülich, Germany

1. Background

1.1. Derivation of mRNA levels

1.1.1 Determining the change in the total mRNA level

We aimed to simulate the impact of DNAzyme treatment on mRNA degradation. For that, the concentration and kinetics of mRNA formation and degradation and the concentration and activity of the DNAzyme in the cell are central factors. To develop a model of the 10-23 DNAzyme influence on mRNA concentration and kinetics, first, we reproduced the kinetic model for 254 genes by Rabani *et al.*[1] and, subsequently, added terms for DNAzyme-mediated mRNA degradation and DNAzyme degradation.

The dynamic change of the total mRNA level X of a given gene can be described by a set of first-order differential equations[1]. X is the sum of the pre-mRNA level P and mature mRNA level M (eq. S1).

$$X = P + M \qquad \text{(eq. S1)}$$

For a description via first-order differential equations, it was assumed that the mRNA of the gene X undergoes three steps. Initially, it is transcribed as pre-mRNA at a rate α, followed by processing into mature mRNA at a rate γ (eq. S2). The mature mRNA is then exported to the cytoplasm, where it is degraded, as determined by the degradation rate β (eq. S3).

$$\frac{dP}{dt} = \alpha(\mathrm{t}) - \gamma(t)P \qquad \text{(eq. S2)}$$

$$\frac{dM}{dt} = \gamma(t)P - \beta(t)M \qquad \text{(eq. S3)}$$

Integrating eqs. S2 and S3 into eq. S1 yields a first-order differential equation to describe the change of the total mRNA level dX over time (eq. S4).

$$\frac{dX}{dt} = \alpha(t) - \beta(t)M \qquad \text{(eq. S4)}$$

The relation between the change in the total mRNA level dX and the current total mRNA level X can be obtained by solving for the change over time, dt (eq. S5); assuming that during a short labeling time β = 0 and all rates are constant, the mature mRNA M can be replaced for the total mRNA X in eq. S4.The resulting equations are integrated using the substitution method (eq. S6).

$$\frac{dX}{dt} = \alpha(t) - \beta(t)X \Rightarrow dt = \frac{dX}{\alpha(t) - \beta(t)X} \quad \text{(eq. S5)}$$

$$\int_0^X \frac{dX}{\alpha(t) - \beta(t)X} = \int_0^t dt \Longrightarrow -\frac{1}{\beta(t)} \int_0^u \frac{du}{u} = \int_0^t dt \quad \text{(eq. S6)}$$

$$with\ u = \alpha(t) - \beta(t)X\ and\ dX = -\frac{1}{\beta(t)} du$$

1.1.2 Deriving the total mRNA level

The resolving of the integrals and the resubstitution of eq. S6 results in eq. S7, which can be solved for the time *t*.

$$-\frac{\ln(u(X))}{\beta(t)} - \frac{-\ln(u(0))}{\beta(t)} = t - 0 \Longrightarrow \frac{-\ln(\alpha(t) - \beta(t)X)}{\beta(t)} - \frac{-\ln(\alpha(t) - \beta(t) \cdot 0)}{\beta(t)} = t \quad \text{(eq. S7)}$$

Rewriting eq. S7 yields X, the total mRNA level (eqs. S8-S10).

$$\ln(\alpha - \beta X) - \ln(\alpha) = -\beta t \Longrightarrow \ln\left(\frac{\alpha - \beta X}{\alpha}\right) = -\beta t \quad \text{(eq. S8)}$$

$$\frac{\alpha - \beta X}{\alpha} = e^{-\beta t} \Longrightarrow \alpha - \beta X = \alpha \cdot e^{-\beta t} \quad \text{(eq. S9)}$$

$$-\beta X = \alpha \cdot e^{-\beta t} - \alpha \Longrightarrow X = \frac{\alpha \cdot e^{-\beta t} - \alpha}{-\beta} \Longrightarrow X = \frac{\alpha}{\beta}(1 - e^{-\beta t)} \quad \text{(eq. S10)}$$

X is affected by the transcription rate α and the degradation rate β. As such, changes in either of these rates over time can have marked effects on the total mRNA level X (eq. S11).

$$X(t) = \frac{\alpha(t)}{\beta(t)}\left(1 - e^{-\beta(t) \cdot t}\right) \quad \text{(eq. S11)}$$

1.2 Calculating rates

1.2.1 Modeling the transcription rate α via an impulse model

To estimate the transcription rate α, two assumptions are made[1]. First, it is assumed that both α and the degradation rate β remain constant over a short observation period. Second, with a short enough labeling time ($t_L \leq 10$ min), the labeled RNA X_{lab} is mostly nuclear and thus subject to negligible degradation ($\beta = 0$). Despite DNAzymes being able to enter the nucleus and, thus, affect the degradation rate, this usually is negligible for the short labeling times used [1]. These assumptions allow for a simplification of equation eq. S11 using L'Hopital's Rule, which now only depends on the labeling time t_L and the transcription rate α. By measuring the expressed mRNA level through experimental data and selecting an appropriately short labeling time, a simplified equation for α is obtained (eq. S12).

$$\lim_{\beta(t)\to 0} X(t) = \lim_{\beta(t)\to 0} \frac{\alpha(t)}{\beta(t)}\left(1 - e^{-\beta(t)\cdot t}\right) \Rightarrow X_{lab}(t) \xrightarrow{\beta(t)=0} t_L\alpha(t) \Rightarrow \alpha(t) = \frac{X_{lab}(t)}{t_L} \qquad \text{(eq. S12)}$$

Eq. S12 provides initial estimates for the transcription rate α, which can be described by an impulse model based on a six-parameter double-sigmoid function, resulting in a smoothing of the experimental data (eq. S13) [2]. To accurately apply this model to fit a rate (e.g., α) or a mRNA level as a function of the time t, multiple parameters are required. These include h_0, which represents either the average value of the rate or mRNA level prior to any significant change, h_1, which represents the maximum value of the rate or mRNA level, and h_2, which represents the average value of the rate or RNA level after the second transition. The slope rate λ describes the rate of change for both the first and second transitions. Furthermore, t_1 and t_2 represent the times at which the first and second transitions occur.

$$\begin{aligned} &rate(t)\ [or\ mRNA\ level(t)] \\ &= \frac{1}{h_1}\cdot\left(h_0 + (h_1 - h_0)\cdot\frac{1}{1+e^{\lambda(t-t_1)}}\right) \\ &\cdot\left(h_2 + (h_1 - h_2)\cdot\frac{1}{1+e^{-\lambda(t-t_2)}}\right) \end{aligned} \qquad \text{(eq. S13)}$$

The impulse model was used to obtain values for both α and X from the data provided by Rabani *et al.* as shown in the Supplementary Table 1 and 2 for all genes[1].

Supplementary Table S1. Experimental total mRNA levels and time provided by Rabani *et al.*[1], the referenced total mRNA levels (using eq. S34 with a scaling factor of 1) as well as the scaled (scaling factor = 0.01 used in eq. S34) and modeled mRNA levels for the gene Socs3. (nd: no data due to computational restraints)

**Time**	**Experimental total mRNA (arbitrary units)**	**Referenced total mRNA (RNA·cell^{-1})**	**Scaled total mRNA (RNA·cell^{-1})**	**Modeled total mRNA (RNA·cell^{-1})**
0	286	3619	36	nd
0	236	2977	30	nd
15	1317	16652	167	168
30	2754	34813	348	372
30	2801	35401	354	372
45	3200	40443	404	373
45	3284	41516	415	373
60	2400	30341	303	305
75	2296	29026	290	267
75	2174	27474	275	267
90	2037	25754	258	257
105	2186	27626	276	255
120	2072	26189	262	255
135	1965	24838	248	255
150	1982	25053	251	254
165	1759	22238	222	254
180	2027	25626	256	nd

Supplementary Table S2. The time, referenced transcription rate α, the transcription rate α of the scaled mRNA levels, as well as the modeled transcription rate α for the gene Socs3.

Time	Referenced α (RNA·cell^{-1}·min^{-1})	Scaled α (RNA·cell^{-1}·min^{-1})	Modeled α (RNA·cell^{-1}·min^{-1})
0	210	2	3
0	352	4	3
15	18785	188	188
30	4103	41	67
30	4219	42	67
45	7870	79	67
45	10626	106	67
60	6028	60	67
75	9020	90	67
75	2881	29	67
90	11186	112	67
105	6514	65	67
120	5633	56	67
135	6624	66	67
150	6091	61	67
165	4779	48	67
180	8407	84	67

1.2.2 Deriving the degradation rate β

The obtained values were then used to calculate the degradation rate β (eq. S14, derived from eq. S5). This requires determining dX/dt, which is the slope of the mRNA level X as a function of the time from the impulse model.

$$\beta(t) = \frac{1}{X} \cdot \left(\alpha(t) - \frac{dX}{dt}\right) \qquad \text{(eq. S14)}$$

Using α, X, and β thus obtained, it is possible to expand the model to incorporate the processing rate γ (eq. S15) and adjust α so that it accounts for the degradation of mRNA when applying longer labeling times. To achieve this, two additional assumptions regarding the rates are made. First, it is assumed that the pre-mRNA is at a steady state during the labeling period, resulting in a value of zero for dP/dt. Second, it is assumed that all other rates (β and γ) remain approximately constant during this period.

$$\frac{dP}{dt} = \alpha(\mathrm{t}) - \gamma(t)P = 0 \qquad \Rightarrow \gamma(t)P = \alpha(\mathrm{t}) \qquad \text{(eq. S15)}$$

This allows the description of the change of the level of mature mRNA over time by replacing the term of $\gamma(t)P$ of eq. S15 with α calculated from eq. S12, resulting in eq. S16:

$$\frac{dM}{dt} = \gamma(t)P - \beta(t)M = \alpha(\mathrm{t}) - \beta(t)M \qquad \text{(eq. S16)}$$

Incorporating the previously obtained β values (eq. S14) as well as the labeling time t_L allows for a new description of the mature mRNA level M (instead of the total mRNA X when using the total time t) without the processing rate γ but instead using the degradation-adjusted transcription rate α_{tl} (eq. S17). This can be solved for α_{tl}, which allows the description of it while also accounting for the degradation of mRNA during labeling. As for the previously calculated α, an impulse model was used for refinement.

$$M(t) = \frac{\alpha_{tl}(t)}{\beta(t)}\left(1 - e^{-\beta(t)\cdot t_L}\right) \qquad \Rightarrow \alpha_{tl}(t) = M(t) \cdot \frac{\beta(t)}{1 - e^{-\beta(t)\cdot t_L}} \qquad \text{(eq. S17)}$$

1.2.3 Obtaining the processing rate

By assuming β = 0 during the labeling period and that all other rates (α and γ) remain constant, it is possible to calculate the processing rate γ via eq. S18.

$$\frac{dP}{dt} = \alpha(t) - \gamma(t)P \qquad \text{(eq. S18)}$$

This results in simplified equations for both the total (eq. S19) and pre-mRNA level (eq. S20), both depending on the time t.

$$\frac{dX}{dt} = \alpha(t) - \beta(t)M = \alpha(t) \qquad \Rightarrow X(t) = \alpha(\mathrm{t}) \cdot t \qquad \text{(eq. S19)}$$

$$\frac{dP}{dt} = \alpha(t) - \gamma(t)P \qquad \Rightarrow P(t) = \frac{\alpha(\mathrm{t})}{\gamma(t)} \cdot \left(1 - e^{-\gamma(t)t}\right) \qquad \text{(eq. S20)}$$

Dividing eq. S20 with eq. S19 yields an equation that only contains the processing rate γ and time t as variables (eq. S21).

$$\frac{P(t)}{X(t)} = \frac{1 - e^{-\gamma t}}{\gamma t} \qquad \text{(eq. S21)}$$

Isolating γ requires multiple steps and results in eq. S32, where γ can be calculated by the total and pre-mRNA level as well as the time t. W is the Lambert W function, also known as the product logarithm, which is the inverse function of $x \cdot e^x$.

These steps are further explained below. First $-e^{-\gamma t}$ needs to be isolated, resulting in eq. S22.

$$-e^{-\gamma t} = \gamma t\frac{P(t)}{X(t)} - 1 \quad \text{(eq. S22)}$$

Now eq. S22 needs to be multiplied by X(t)/P(t) to obtain eq. S23.

$$-\frac{X(t)}{P(t)} \cdot e^{-\gamma t} = \gamma t - \frac{X(t)}{P(t)} \quad \text{(eq. S23)}$$

Afterward, $\gamma \cdot t$ can be isolated to eq. S24.

$$-\frac{X(t)}{P(t)} \cdot e^{-\gamma t} + \frac{X(t)}{P(t)} = \gamma t \quad \text{(eq. S24)}$$

Now the exponential term on the left side of the term can be eliminated by multiplying with $e^{\gamma t}$ (eq. S25).

$$-\frac{X(t)}{P(t)} + \frac{X(t)}{P(t)} \cdot e^{\gamma t} = \gamma t \cdot e^{\gamma t} \quad \text{(eq. S25)}$$

This term can be rearranged again, which is shown in eq. S26.

$$\frac{X(t)}{P(t)} \cdot e^{\gamma t} - \gamma t e^{\gamma t} = -\frac{X(t)}{P(t)} \quad \text{(eq. S26)}$$

Now eq. S26 can be simplified to eq. S27 for further usage.

$$e^{\gamma t}\left(\frac{X(t)}{P(t)} - \gamma t\right) = -\frac{X(t)}{P(t)} \quad \text{(eq. S27)}$$

To further use the Lambert W function with γ, eq. S27 needs to be substituted as shown in eq S28.

$$\gamma t - \frac{X(t)}{P(t)} = u \; and \; \gamma = \frac{P(t)u + X(t)}{P(t)t}$$

$$e^{t\frac{P(t)u+X(t)}{P(t)t}}\left(\frac{X(t)}{P(t)} - t\frac{P(t)u + X(t)}{P(t)t}\right) = -\frac{X(t)}{P(t)} \quad \text{(eq. S28)}$$

Now eq. S28 can be simplified to eq. S29.

$$-e^{\frac{P(t)u+X(t)}{P(t)}} u = -\frac{X(t)}{P(t)} \quad \text{(eq. S29)}$$

Eq. S29 needs to rewritten to obtain a form that can be used for the Lambert W function, which results in eq. S30.

$$e^{\frac{P(t)u+X(t)}{P(t)}} u = \frac{X(t)}{P(t)} \Longrightarrow e^{u}u = \frac{e^{-\frac{X(t)}{P(t)}}X(t)}{P(t)} \quad \text{(eq. S30)}$$

When using the Lambert W function on eq. S30, u can be isolated (eq. S31).

$$u = \mathrm{W}\left(\frac{e^{-\frac{X(t)}{P(t)}}X(t)}{P(t)}\right) \qquad \text{(eq. S31)}$$

After resubstituting, the final equation of the processing rate γ can be obtained via eq. S32.

$$\gamma t - \frac{X(t)}{P(t)} = W\left(\frac{e^{-\frac{X(t)}{P(t)}}X(t)}{P(t)}\right) \Rightarrow \gamma = \frac{P(t) \cdot W\left(\frac{e^{-\frac{X(t)}{P(t)}} \cdot X(t)}{P(t)}\right) + X(t)}{t \cdot P(t)} \qquad \text{(eq. S32)}$$

With the new values of α and γ, it is now possible to calculate the level of pre-mRNA P using eq. S20. However, the Lambert W function in eq. S32 can have multiple branches so only the principle branch is used as other branches include imaginary numbers, which are incompatible with real-valued kinetic data. This provides a full description of the different mRNA levels of any gene.

1.3 Normalization and conversion of raw data for kinetic modeling of mRNA levels

To recalculate the kinetic models for each gene published by Rabani *et al.*[1] based on the equations discussed in the previous chapter, the measured mRNA levels over time as provided in their supplements were used. However, the reported values (Supplement table 2, columns B and AL-BS in [1]) were normalized to a standard internally set in the measurement and given in arbitrary units due to the provided data being readouts of the nCounter System used by Rabani *et al.*[1]. As the equations require the use of absolute concentrations and the effectiveness of the DNAzyme depends on the substrate concentration, the normalized units had to be translated to absolute concentrations. Therefore, the peak of the different mRNA levels (X, P, and M) of each gene was normalized and afterward scaled to represent the average concentration of the total mRNA[2, 3] in a cell (see below).

Although this likely results in higher than usual mRNA levels for the investigated genes, one of the intended targets for the application of DNAzymes is viral RNA as well as overexpressed proteins, which both can reach high mRNA levels in cells. Furthermore, for later applications, the experimentally determined levels and kinetics of mRNA of target

genes can be used to calculate a kinetic model and determine the DNAzyme influence on a per-case basis.

Normalization was done by dividing each data point of an mRNA level by the highest data point of that mRNA level for the respective gene; the data points were then scaled by multiplying the normalized values with the average concentration of mRNA in a cell. The average amount of mRNA in a cell is in the range of 0.1-0.2 pg [4, 5]. Combined with the average nucleotide count of 2,000 nts, which corresponds to a molecular weight of 641,159 g/mol[6], this results in 187,851 mRNA molecules per cell when assuming 0.2 pg (eq. S33), which fits well with the experimentally determined count of 200,000 mRNA molecules within a mammalian cell[7]. However, it should be noted that up to 0.5 pg mRNA per cell was reported for some cell types, which may need to be considered for highly transcribing cell types [8].

$$molecules_{mRNA} = \frac{0.2\,pg}{641{,}159\,\frac{g}{mol}} \cdot 6.022 \cdot 10^{23}\,\frac{molecules}{mol} \approx 187{,}851 \qquad \text{(eq. S33)}$$

The absolute mRNA level is then obtained with eq. S34, where d is the normalized data ($\{\, d \in \mathbb{R} \mid 0 \leq d \leq 1\}$) and s = 0.01 is a scaling factor introduced to reflect the transcription of a single gene instead of the entire cell (see also chapter Determining the change in the total mRNA level for its influence), which is considered the targeted gene of the DNAzyme. This resulted in values that are concordant with those for highly expressed genes, showing the effect of the DNAzyme during a high mRNA concentration[9].

$$mRNA\ level = d \cdot 187{,}851 \cdot s \qquad \text{(eq. S34)}$$

2. Methods

2.1 Calculation of rates

The previously described calculations in chapter 0 were implemented in Python 3.9; the script is provided as Supplement 2. The script uses the *pandas* and *csv* libraries for importing and exporting data, respectively. The *warnings* library was employed to ease debugging and identify potential issues.

For numerical calculations, the *numpy* library was used with the *scipy.special* module to import the Lambert W function. The *curve_fit* and *differential_evolution* modules from the *scipy* library were imported to generate and fit parameters to the mRNA levels and rates. Finally, the *stats* function of *scipy* was used to perform error calculations, which are listed in Supplement 3.

To test the model, we used the data provided in Supplementary Table 2 by Rabani *et al.*[1] transformed to absolute concentrations as shown in eq. S34. To calculate α via eq. S12, data with a labeling time of ten minutes is required (columns AL-BS). It was assumed that the labeling marks all newly produced mRNA and, as such, describes the newly transcribed mRNA before any processing. The labeled mRNA fraction of the experiment accounts for nuclear mRNA and is not subjected to any relevant degradation, thus, representing the pre-mRNA level P over time. After transforming the data, the mature mRNA level was calculated by subtracting the labeled mRNA from the total mRNA X (eq. S1).

After obtaining α (eq. S12), it was plotted against the time. To smooth the experimental data, an impulse model (eq. S13) was used to fit α for all provided genes, which was optimized by using differential evolution with the Latin hypercube algorithm. To fit the rates or mRNA levels, a varying slope rate is used while imposing bounds, ensuring that data falls within reasonable physical values. For the second transition point, a minimum time of 17.5 minutes is set to avoid similarity with the first transition point, which can lead to the model becoming stuck in a local minimum. In the initial differential evolution evaluation, the slope rate begins at -0.02, decreases by 0.02, and stops at -10. Subsequently, these parameters are assessed using *curve_fit* with the slope rate bounds doubled and the minimum time of the second transition bound reduced to 0.0001 to enable optimization in either direction if necessary. In instances where negative slope rate parameters are unattainable, a new step is initiated to search for positive slope rates,

ranging from 0.005 to 2.5 with 0.005 increment steps. If positive slope rates are still unobtainable, a broader search is initiated with a starting point of 0.1, incrementing by 0.1, and concluding at 50. For each step, the goodness of the fit was determined by R, R^2, the standard deviation, and the root mean square error, when the parameters were optimized for high R values. Additionally, a reduced χ^2 was calculated and used in the optimization to determine if either this or R is a more favorable criterion for modeling the mRNA levels. Both statistics and calculated values are provided in Supplement 3, which can also be obtained by running the python script provided as Supplement 2.

The fitting was repeated for the experimental total mRNA level X. Values of dX/dt from eq. S4 were determined by calculating the slope of the fitted model for $X(t)$. The data of the fits, as well as the obtained dX/dt values, were then used to calculate β using eq. S14. Using β, it is also possible to describe the transcription rate α_{tl} while considering the degradation of mRNA during labeling times longer than ten minutes. To account for the mRNA degradation during labeling, eq. S17was used to calculate new values of α. This new estimate of α was compared to the experimental data to test how well it describes the data (see Supplement 2 and 3). To smooth the values of the new α, another impulse model, as shown in eq. S13, was fitted.

To calculate the processing rate γ, the pre-mRNA level P needs to be fitted with an impulse model (eq. S13). Afterward, γ can be calculated using eq. S32 with the fitted pre-mRNA and the fitted total mRNA level. The mature and pre-mRNA levels can be calculated by inserting the obtained rates into eqs. S3 and S2, respectively.

2.2 Including DNAzyme-mediated mRNA degradation

To expand the kinetic model for considering the activity of a DNAzyme, a pseudo-first-order kinetic model was chosen according to which the DNAzyme concentration is considered constant compared to the mRNA level changes, which show rapid changes due to transcription and degradation. First, the rate constant k_{DZ} that describes the mRNA degradation by the DNAzyme is calculated from the experimentally observed rate k_{obs} and the DNAzyme concentration [DZ] (eq. S35). Borggräfe *et al.*[10] measured these values for a 10-23 DNAzyme variant (abbreviated as A5C-DZ) (A5C-DZ: $k_{\text{obs}} = 7.32 \cdot 10^{-4}$ s^{-1}; [DZ] = $0.4 \cdot 10^{-6}$ M) and a mutant with thioguanine instead of guanine at position 14 of the catalytic loop (Thio-DZ: $k_{\text{obs}} = 4.16 \cdot 10^{-3}$ s^{-1}; [DZ] = $0.4 \cdot 10^{-6}$).

$$k_{DZ} = \frac{k_{obs}}{[DZ]_{rate}^{m}} \quad \Rightarrow \quad k_{WT-DZ} = \frac{7.32 \cdot 10^{-4}\, s^{-1} \cdot 60\, \frac{s}{min}}{0.4 \cdot 10^{-6}\, M} = 109{,}800\, min^{-1} M^{-1} \qquad \text{(eq. S35)}$$

Since a single DNAzyme cleaves one RNA molecule per reaction, *m* is 1. The rate constant k_{DZ} was calculated for both A5C-DZ (eq. S35) and Thio-DZ ($k_{Thio\text{-}DZ}$ = 624,000 min^{-1} M^{-1}); k_{DZ} values were also increased by factors of 100 and 1000 to simulate more effective DNAzyme variants.

With this information, the calculation of *dX/dt* (eq. S4) is expanded to include the effect of DNAzyme-mediated mRNA degradation. The right-most term in eq. S36 describes the effect of the DNAzyme on the mRNA level of a targeted gene and depends on [*DZ*], k_{DZ}, the time *t*, the half-life of the DNAzyme $T_{1/2}$, and the level of mature mRNA *M* obtained by inserting the referenced data of eq. S14 into eq. S1. For A5C-DZ, a half-life of 70-80 min in human serum and plasma and approximately 2 h in fetal calf serum was reported[**Error! Reference source not found.**, 11]. For applications of not-stabilized DZ we, therefore, here chose a DNAzyme half-life of 75 minutes for all calculations. Note that this half-life is a conservative estimate and more biostable variants have already been developed; further improving the cellular performance on longer time scales (see Fig. S2).

$$\frac{dX}{dt}_{DZ} = \alpha(t) - \beta(t)M - [DZ] \cdot k_{DZ} \cdot 0.5^{\frac{t}{T_{1/2}}} \cdot M \qquad \text{(eq. S36)}$$

With the adjusted mRNA level change over time, it is now possible to recalculate β via eq. S14 resulting in eq. S37.

$$\beta(t)_{DZ} = \frac{1}{X} \cdot \left(\alpha(t) - \frac{dX}{dt}_{DZ}\right) \qquad \text{(eq. S37)}$$

$\beta(t)_{DZ}$ is inserted into eq. S4 to obtain the time course of the total mRNA level $X(t)_{DZ}$ in the presence of the DNAzyme (eq. S38).

$$X(t)_{DZ} = \frac{\alpha(t)}{\beta(t)_{DZ}}\left(1 - e^{-\beta(t)_{DZ} \cdot t}\right) \qquad \text{(eq. S38)}$$

3. Results

3.1 Modeling mRNA kinetics

A Pearson correlation coefficient of R > 0.01 was used as a criterion for a good fit of the experimental kinetics of mRNA levels according to eq. S13 as this was also used by Rabani *et al.*[1] We obtained R > 0.01 for 97% (247/254) of the 254 genes provided by Rabani *et al.* [1] when optimizing the model according to R (Fig. S5). Genes for which the modeling yielded an R > 0.95 (33%, 85 of 254 provided genes) were considered adequately modeled (Fig. Ss1A). Compared to this, the optimization with a reduced χ^2 test (using 8 degrees of freedom: 6 variables are used for the impulse model and a total of 14 points are fitted for the mRNA model; eqs. 39 and 40; see also Supplement 2)

$$\chi^2 = \sum \frac{(Model - referenced\ Data)^2}{referenced\ Data} \qquad \text{(eq. S39)}$$

$$reduced\ \chi^2 = \frac{\chi^2}{Degrees\ of\ freedom\ (8)} \qquad \text{(eq. S40)}$$

yielded R > 0.01 for 96% (243/254) of the genes and R > 0.95 for 33% (84 of 254) of the genes (Fig. S1B). Overall, both optimization approaches allow modeling most of the genes but suffer from noisy experimental data or sudden, large changes in the mRNA level. We chose the data from optimizing R for further analyses. For comparison, Rabani *et al.* [1] successfully modeled 92% (233/254) of the genes with R >0.01.

For some genes, it is difficult to discern whether the modeling of the experimental data is unsatisfying or if the experimental data itself lacks a definitive pattern (Figure S1C). This ambiguity makes it challenging to accurately assess the quality of the model for such genes.

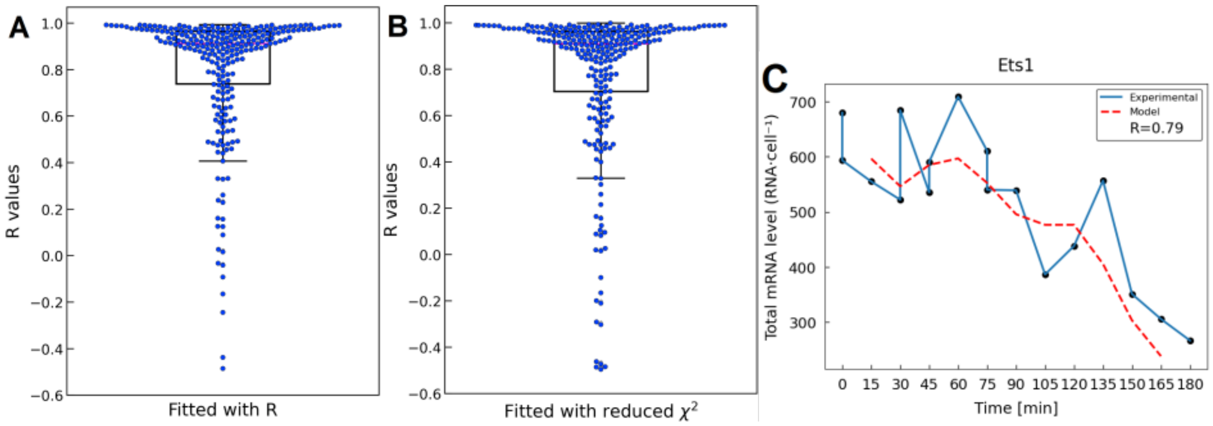

Figure S1: Accuracy evaluation of modeling mRNA kinetics. **A**: Boxplot of R values (blue) of the final model for all genes using R as a criterion to optimize the model. The median is 0.912 (magenta); the upper and lower box lines describe the interquartile range and the whiskers the range of the R values. **B**: Boxplot of R values (blue) of the final model for all genes using the reduced χ^2 as a criterion to optimize the model. The median is 0.910 (magenta); the upper and lower box lines describe the interquartile range and the whiskers the range of the R values. **C**: Example of the time course of the total mRNA level for gene Ets1, for which the model described in chapter 2.2 yields R = 0.79. Due to the large variations in the experimental data, it is difficult to fit such data with the kinetic model. The variations could be a result of experimental uncertainties as suggested for the data points at 30 and 75 minutes.

3.2 Effect of DNAzyme concentration and cleavage rate

In the development of new DNAzymes, key factors for high activity are the cleavage rate and the maximum concentration reachable within a cell by the chosen delivery system. Maximizing the activity is desirable to reduce the required dosage, minimizing the burden of high on-target concentrations, and particularly enabling an effective treatment. To estimate the impact of these parameters, the model of RNA levels was expanded as shown in equations eqs. S36-38. Figure S2 exemplarily shows the effect of different DNAzyme concentrations and cleavage rates on the mRNA levels of the gene Socs3 according to eq. S38.

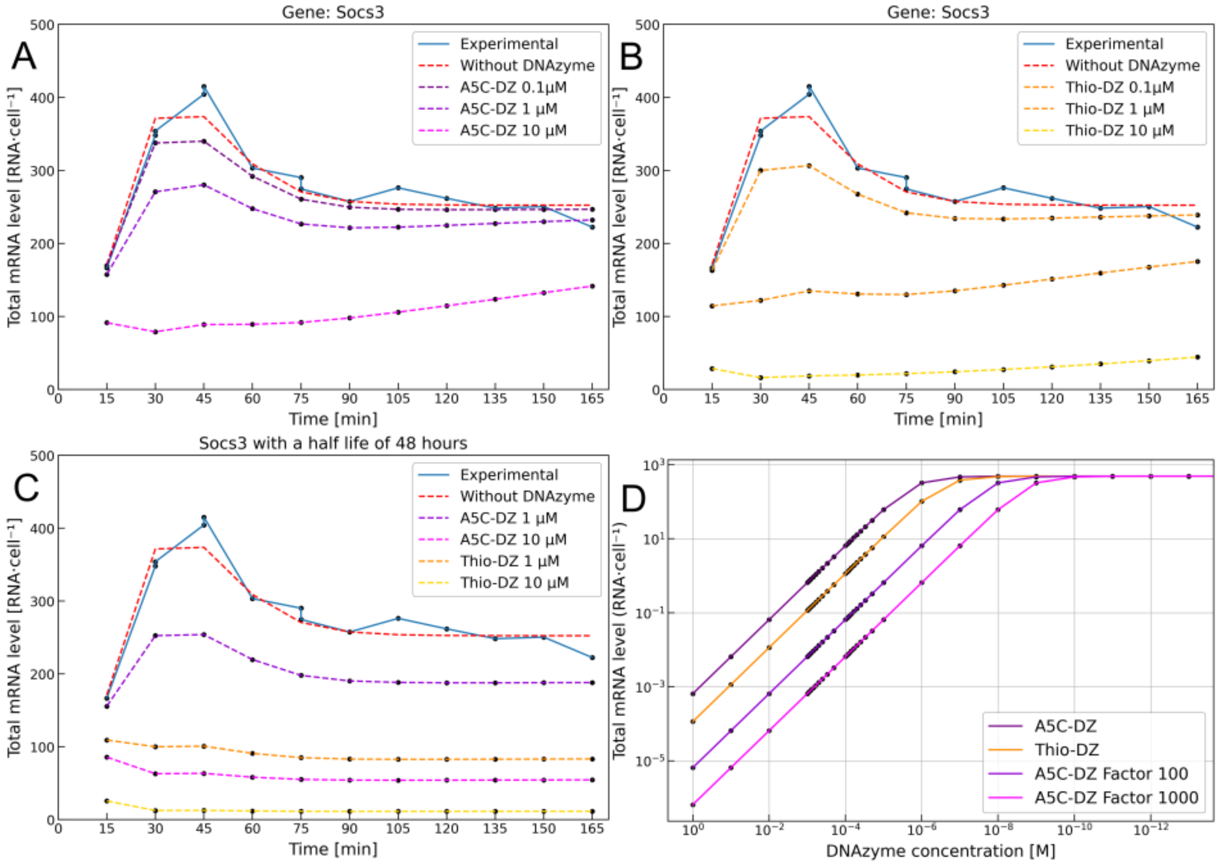

Figure S2: Effect of different concentrations and cleavage rates of A5C-DZ and Thio-DZ on the Gene Soc3. The mRNA level increases after ~75 min due to the degradation of the DNAzyme with a half-life of 75 minutes. **A**: Effect of 0.1, 1, and 10 µM A5C-DZ on the time course of the mRNA level of Socs3. **B**: Effect of 0.1, 1, and 10 µM Thio-DZ on the time course of the mRNA level of Socs3. **C:** Effect of a DNAzyme half life of 48 hours instead of 75 minutes with DNAzyme concentrations of 1 and 10 µM. **D**: The effect of the DNAzyme concentration on the maximum mRNA level of a cell is shown for Socs3 with different cleavage rates.

As expected, an increase in DNAzyme concentration and/or cleavage rate reduces the mRNA levels. For A5C-DZ, a concentration in the range of 1 µM is required to obtain a >25% decrease in the peak level; for Thio-DZ, only 0.1 µM is required. For comparison, a 35% decrease in mRNA levels can already lead to persistent atrial fibrillation,[12] showing the impact a DNAzyme could have as a pharmaceutical agent. As the transcription of most genes highly correlates with the respective protein expression [13, 14], one can assume that the reduction seen in Fig. S2 affects the expression of the protein by a similar amount. In yeast, a correlation of $R = 0.93$ between the mRNA and protein levels was reported, further highlighting the importance of even small mRNA level changes.[15]

An impulse model was used to derive the rates α, β, and γ. This type of model is typically used to, e.g., describe drug levels over time after tablet administration, i.e., when absorption and degradation processes overlap such that a typical time course results as seen for the gene Socs3 (Figure S2). Still, the impulse model and the kinetic model also describe the time courses of the mRNA levels well that deviate from the typical course,

e.g., when the mRNA level sharply rises or falls or when a clearly identifiable plateau is lacking. For such time courses, our current model can also yield fits of the experimental data with $R \geq 0.95$ (Figure S3). This result indicates that the effect of DNAzymes on the mRNA levels of future target genes can also be reliably modeled.

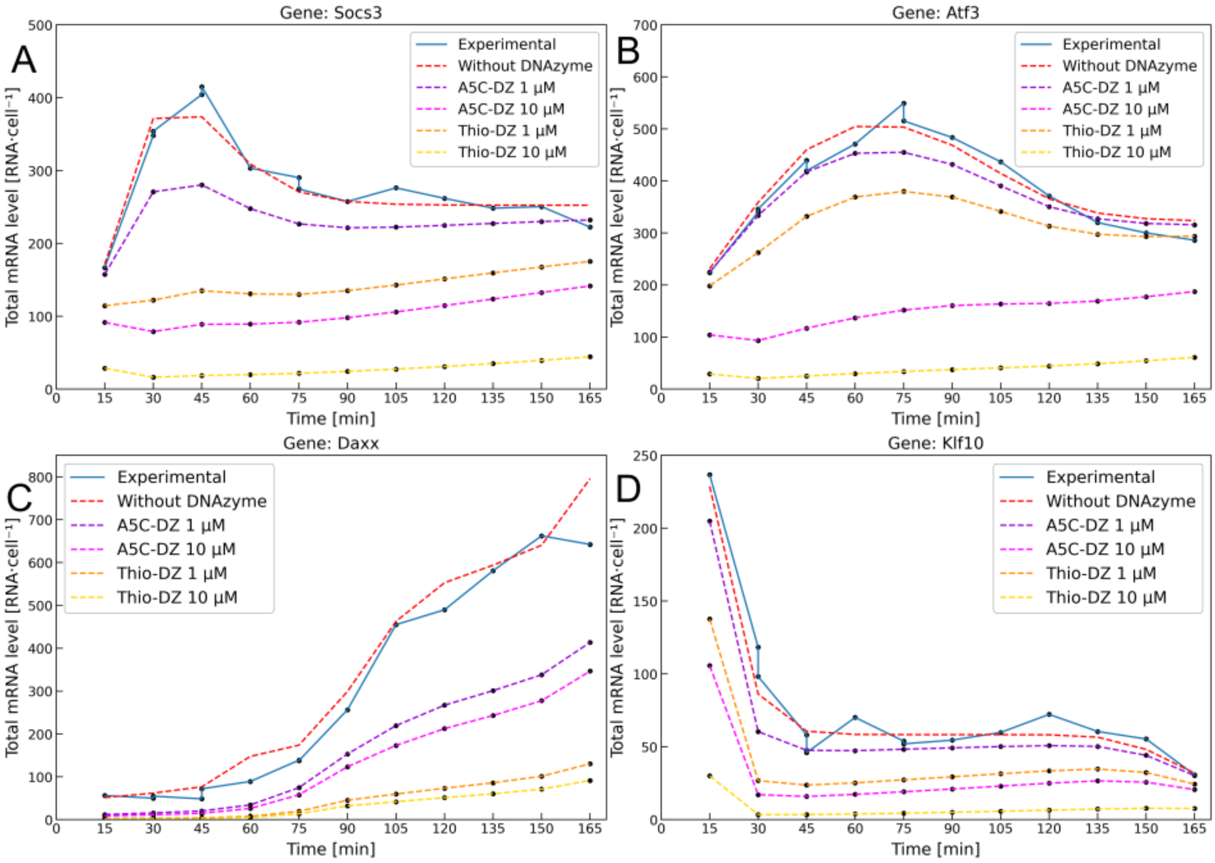

Figure S3: Modeling of selected genes ($R > 0.95$) and the decrease of the mRNA level depending on the concentration of A5C-DZ and Thio-DZ. **A**: A time course with a peak at ~45 minutes and a steady state after ~90 minutes. **B**: A parabola-shaped time course showing a maximum at ~75 minutes. **C**: Steadily rising time course with a possible plateau after ~150 minutes. **D**: Sharply decreasing time course with a steady state after ~45 minutes.

Overall, the model allows for reliable predictions of the time-dependent changes in mRNA levels for genes with different characteristic time courses, providing a promising baseline for further expansion of the model. With more data, it will be possible to compare longer experimental times, different gene sets, and new DNAzymes, allowing better prediction of the properties of potential therapeutic DNAzymes.

3.3 Adjustment of mRNA levels

The scaling factor (eq. S34) in the model determines the absolute levels of mRNA for a given gene and can be varied to simulate different scenarios on target. This approach is useful for adjusting the model quickly for highly expressed mRNA, such as in virus infections or cancer. In Figure S4, the gene Socs3 is shown with the scaling factor

increased ten- and one hundred-fold, which results in similarly scaled time courses of the RNA level if or if no DNAzyme is present as compared to Figure S3A.

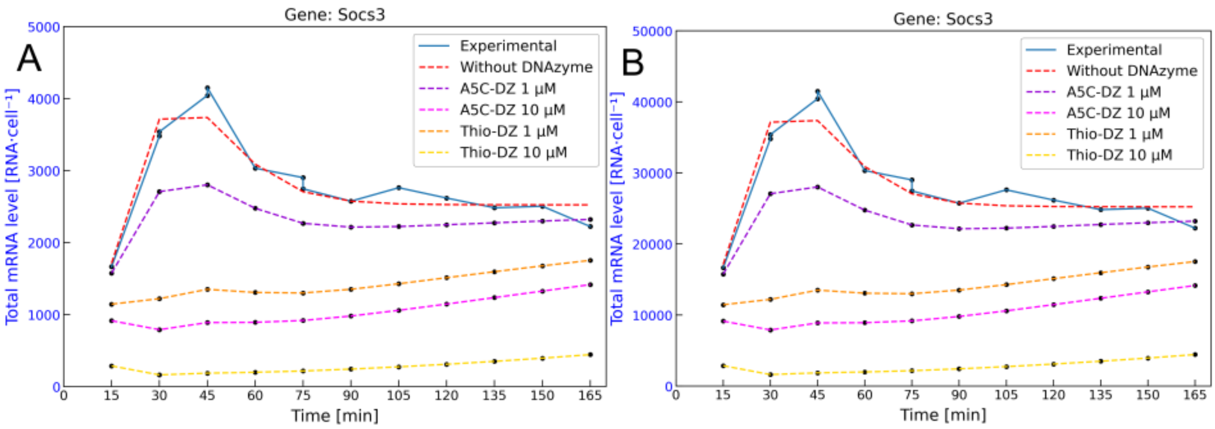

Figure S4: Effect of the scaling factor (eq. S34) on the time course of mRNA levels with the y-axes highlighted in blue to indicate the change in the mRNA levels compared to Fig. S3A. **A**: The mRNA level of gene Socs3 with a ten times higher scaling factor of 0.1. **B**: The mRNA level of gene Socs3 with a hundred times higher scaling factor of 1. In comparison to the original scaling factor of 0.01, similar but scaled time courses of the RNA level are obtained in the presence or absence of DNAzyme.

Currently, adjusting the scaling factor only alters the absolute levels of mRNA but not the relative decrease due to DNAzyme activity. This is due to the change in degradation rate, which is dependent on the mature mRNA level M (eq. S37). With the current concentrations of DNAzyme and mRNA, we can assume that the DNAzyme is always occupied by mRNA, leading to a cleavage rate of the mRNA that is linear in its concentration (eq. S36). This leads to a good description for the chosen time frame of 180 minutes. When expanding the model to longer times and, hence, reduced mRNA concentrations, it might be necessary to describe the DNAzyme action with a Michaelis-Menten kinetics or second-order kinetics to overcome the assumption of a pseudo first-order kinetic. Upon reduced mRNA concentrations, there are far more DNAzymes than mRNAs, leading to an exponential decay in the mRNA concentration rather than a linear one as assumed under first-order kinetics.

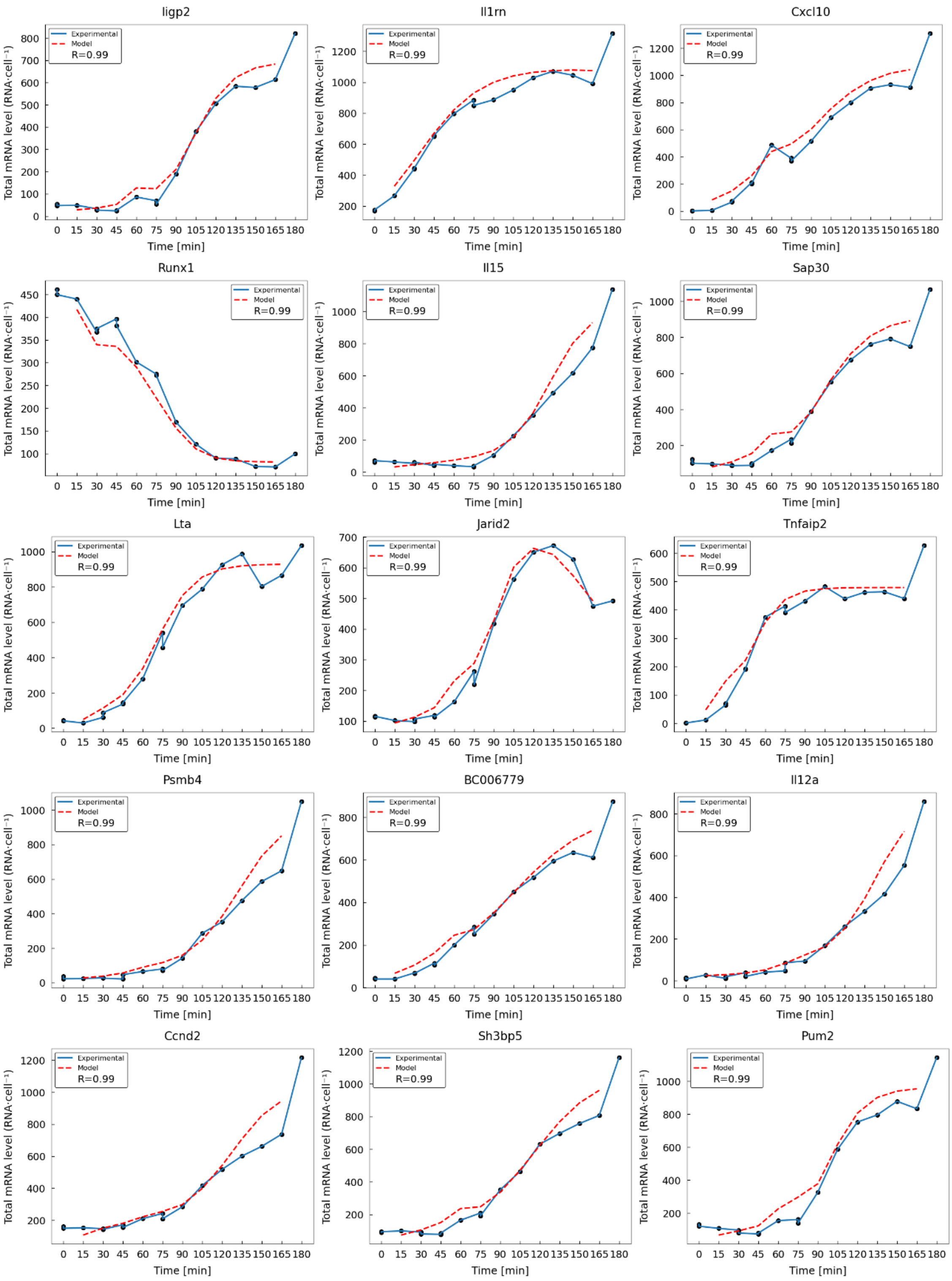

Figure S5: All 254 genes were fitted by maximizing *R*; they are sorted by *R*, starting with the highest *R* values. Continued on Pages 20-35.

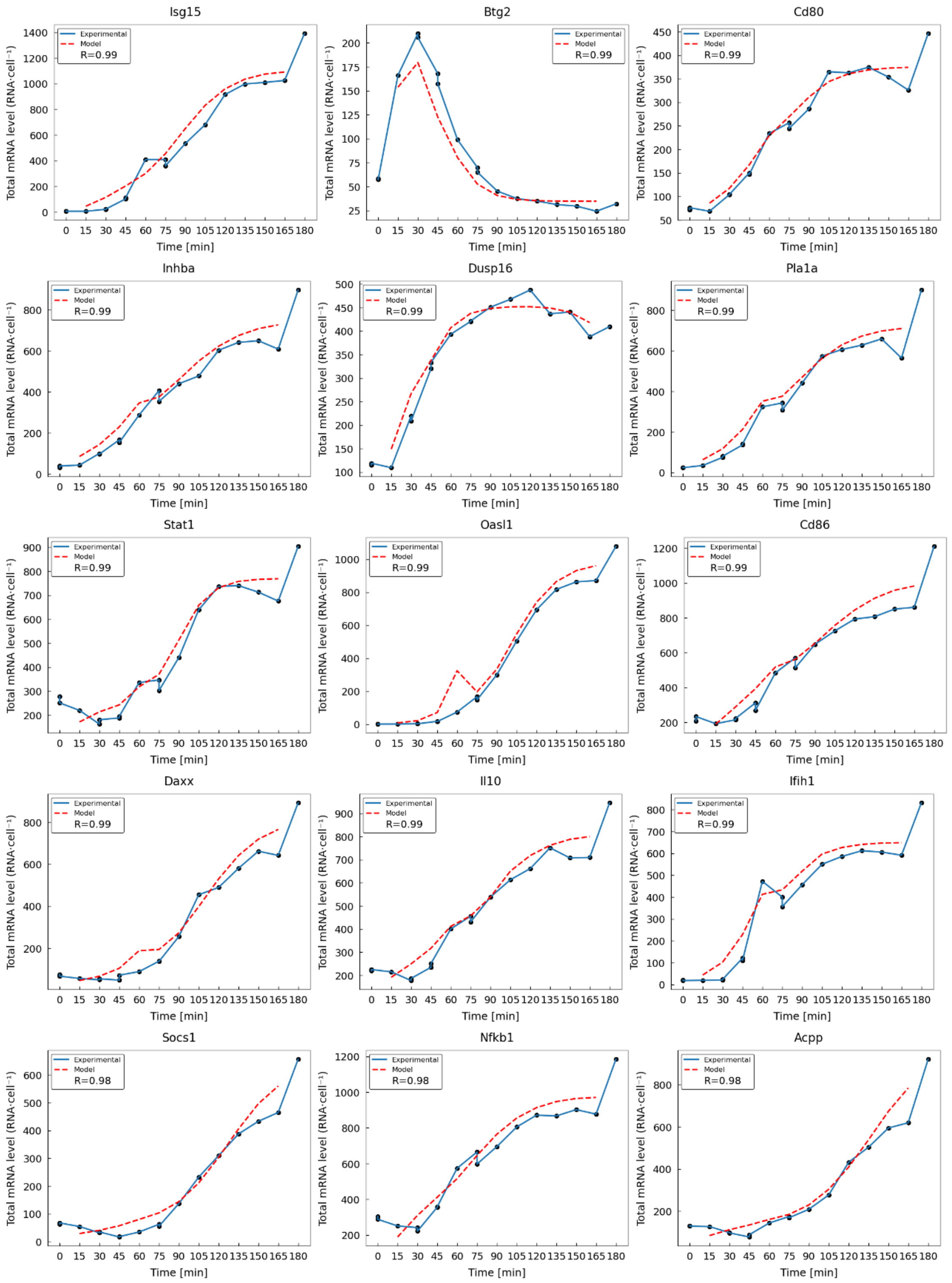

Figure S5 continued.

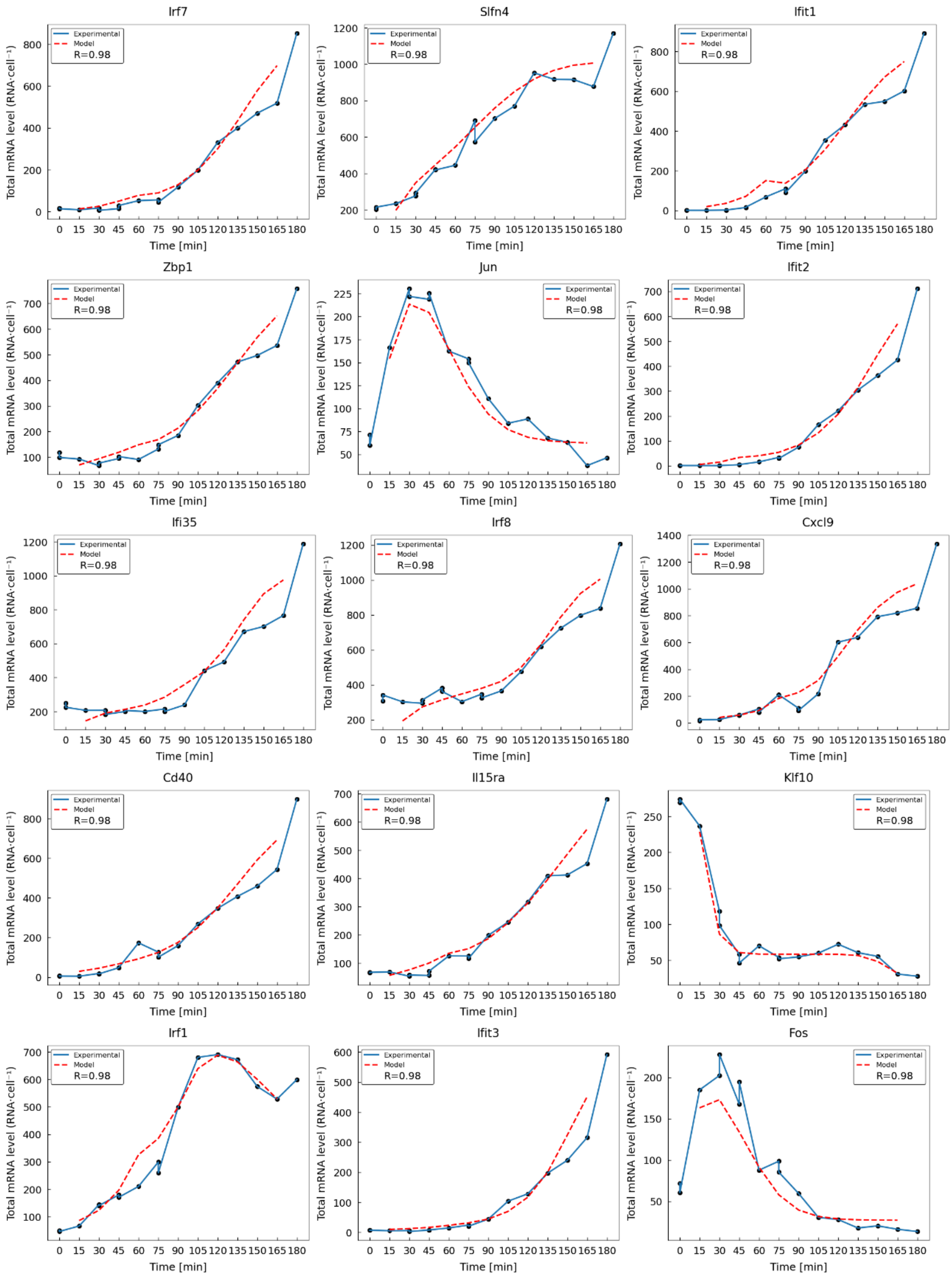

Figure S5 continued.

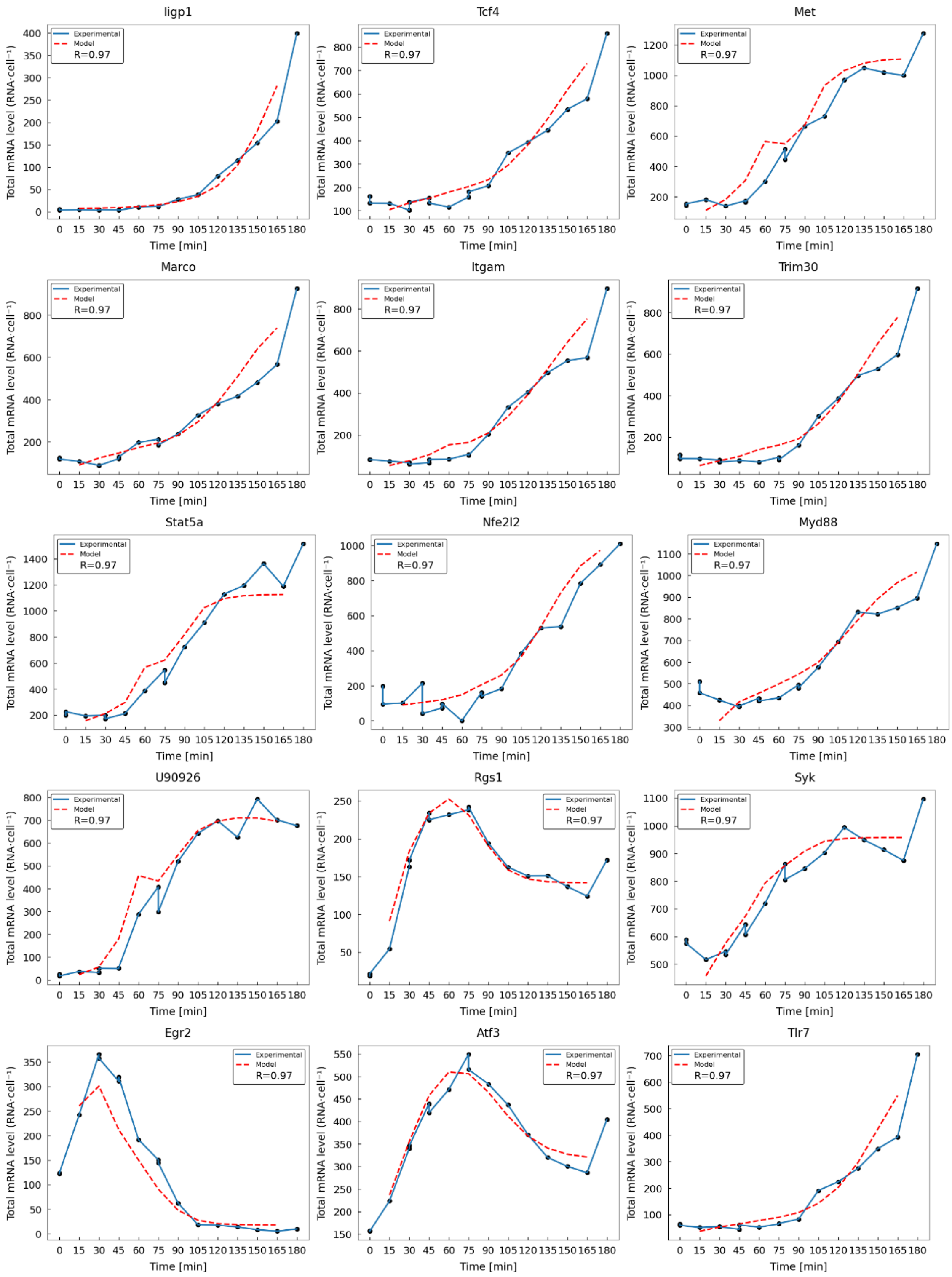

Figure S5 continued.

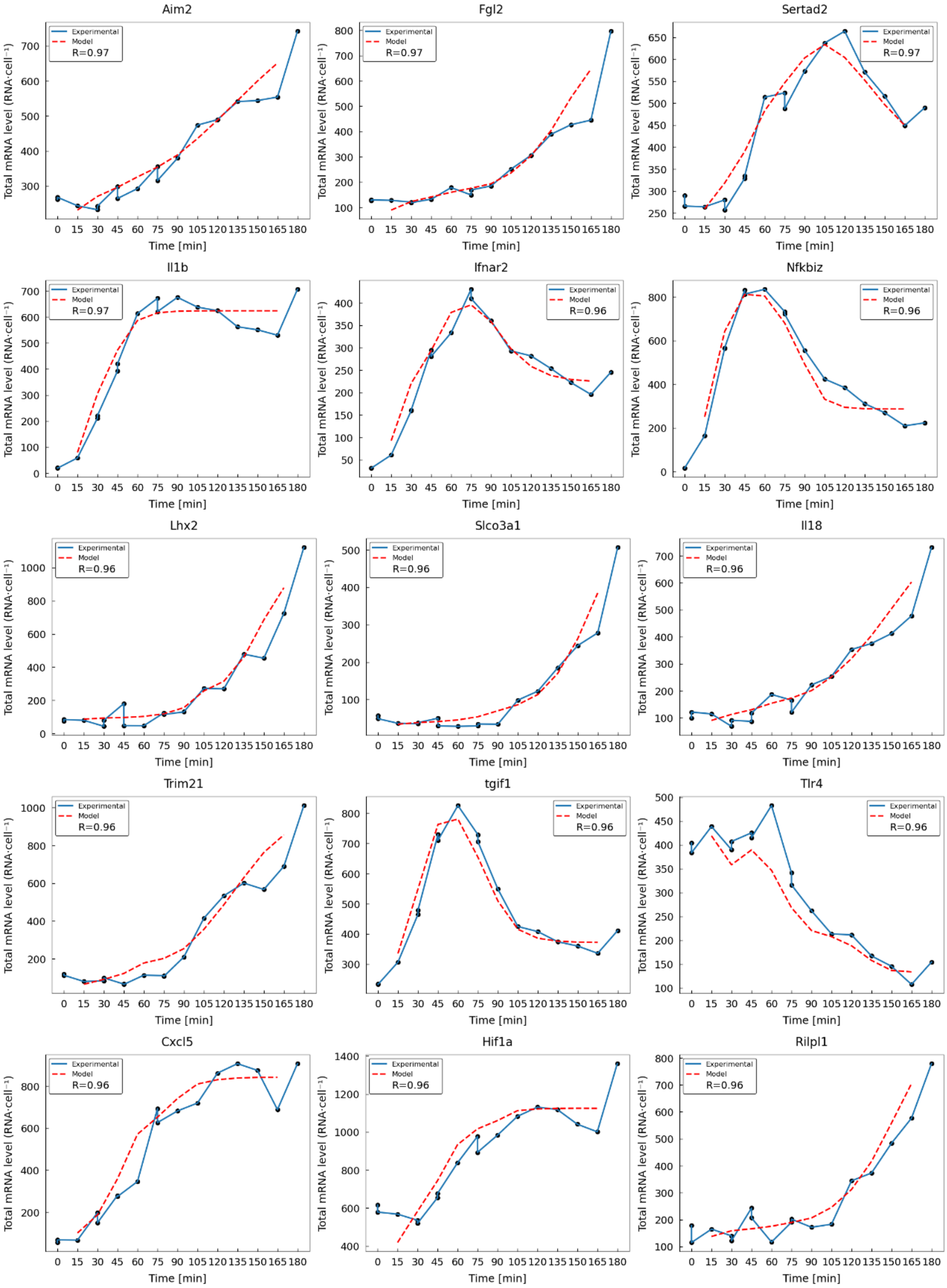

Figure S5 continued.

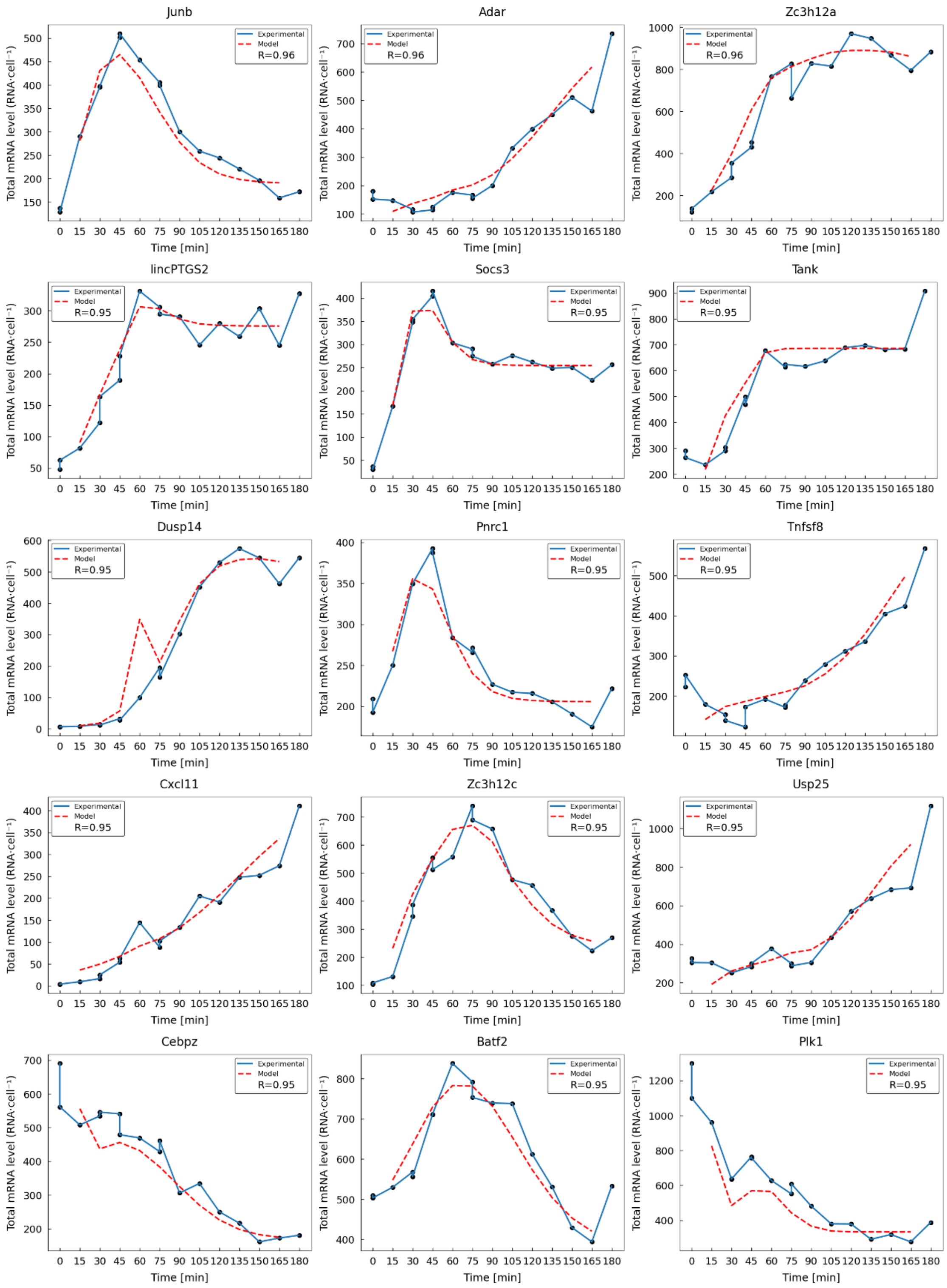

Figure S5 continued.

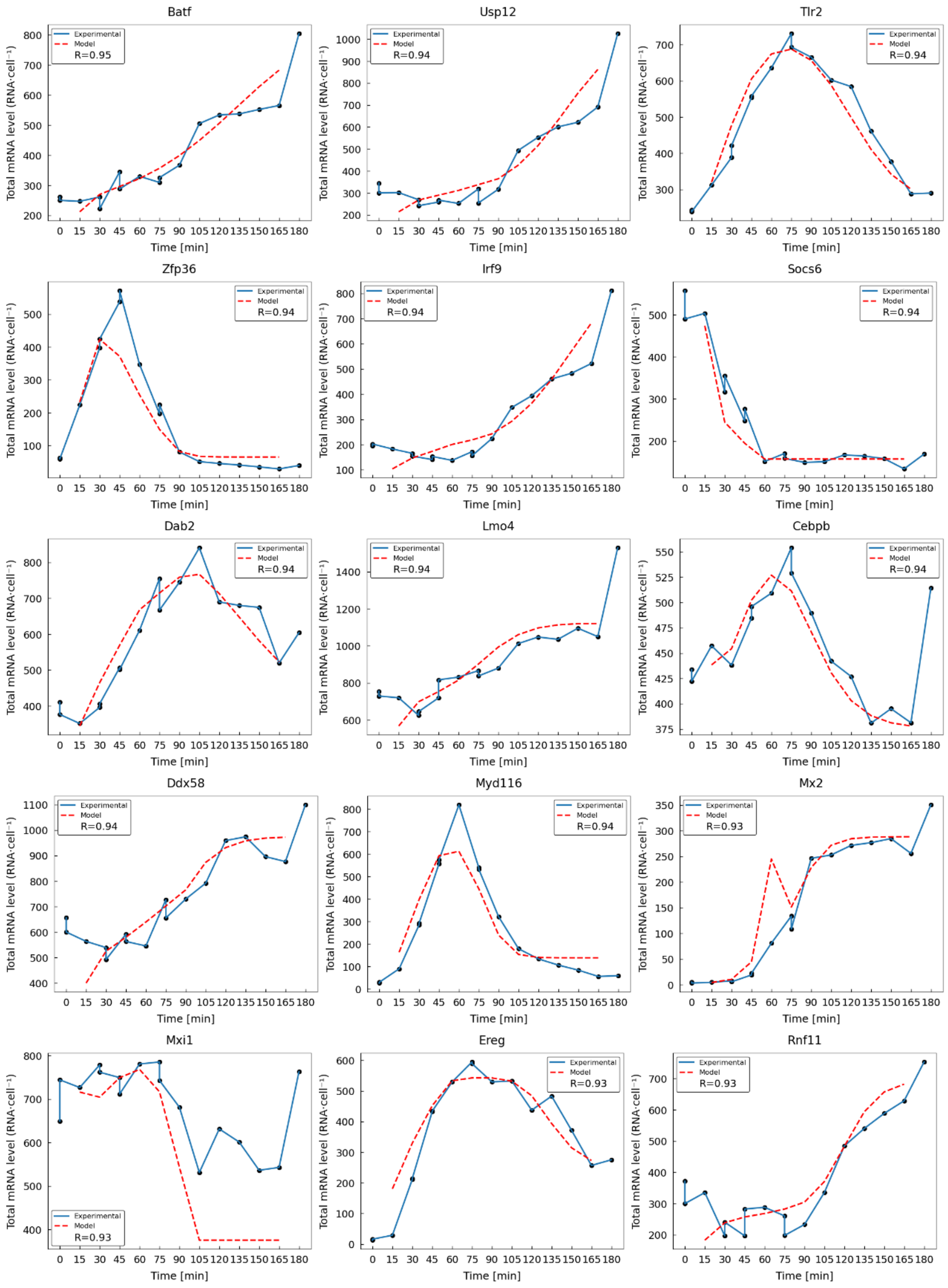

Figure S5 continued.

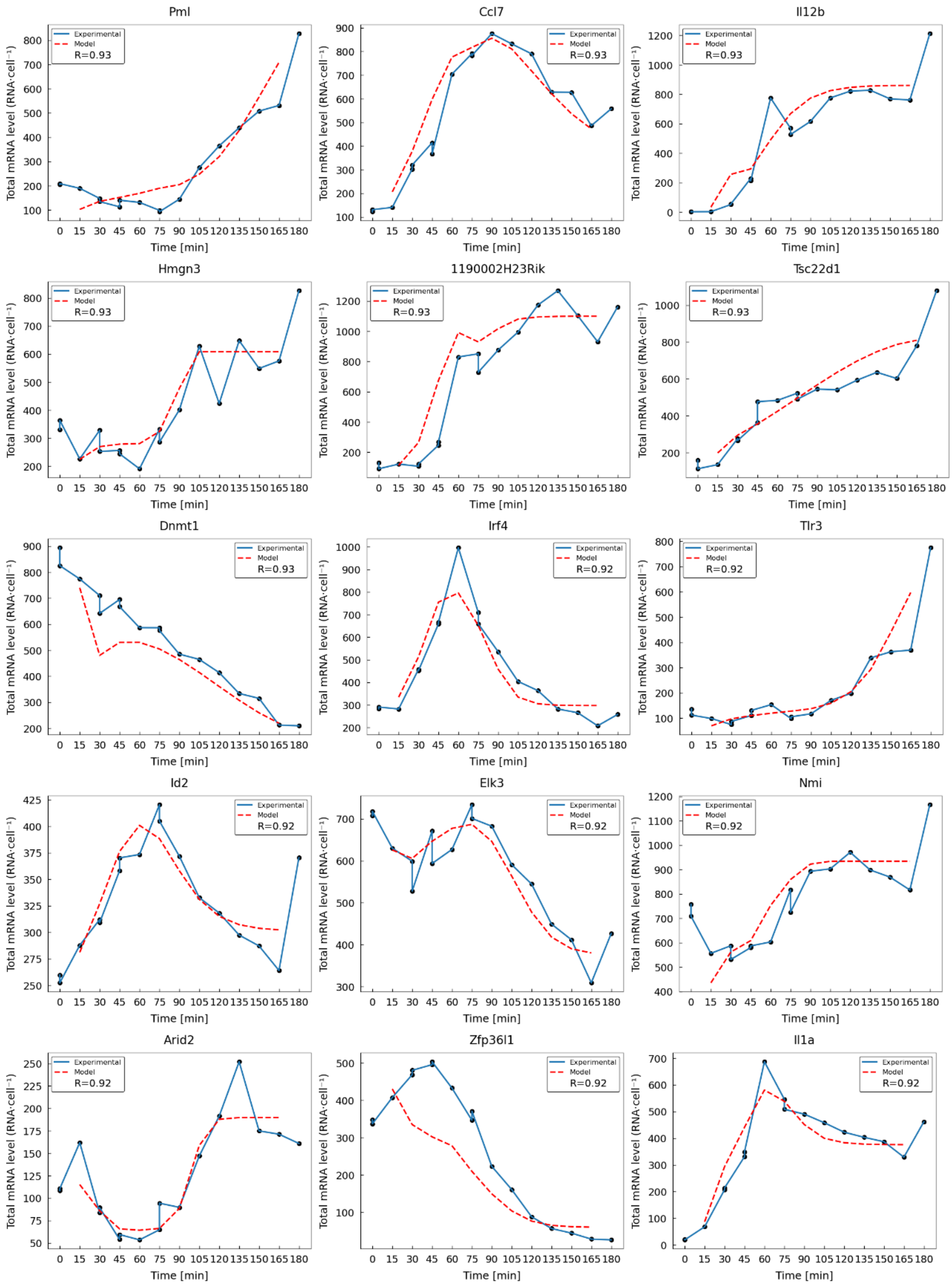

Figure S5 continued.

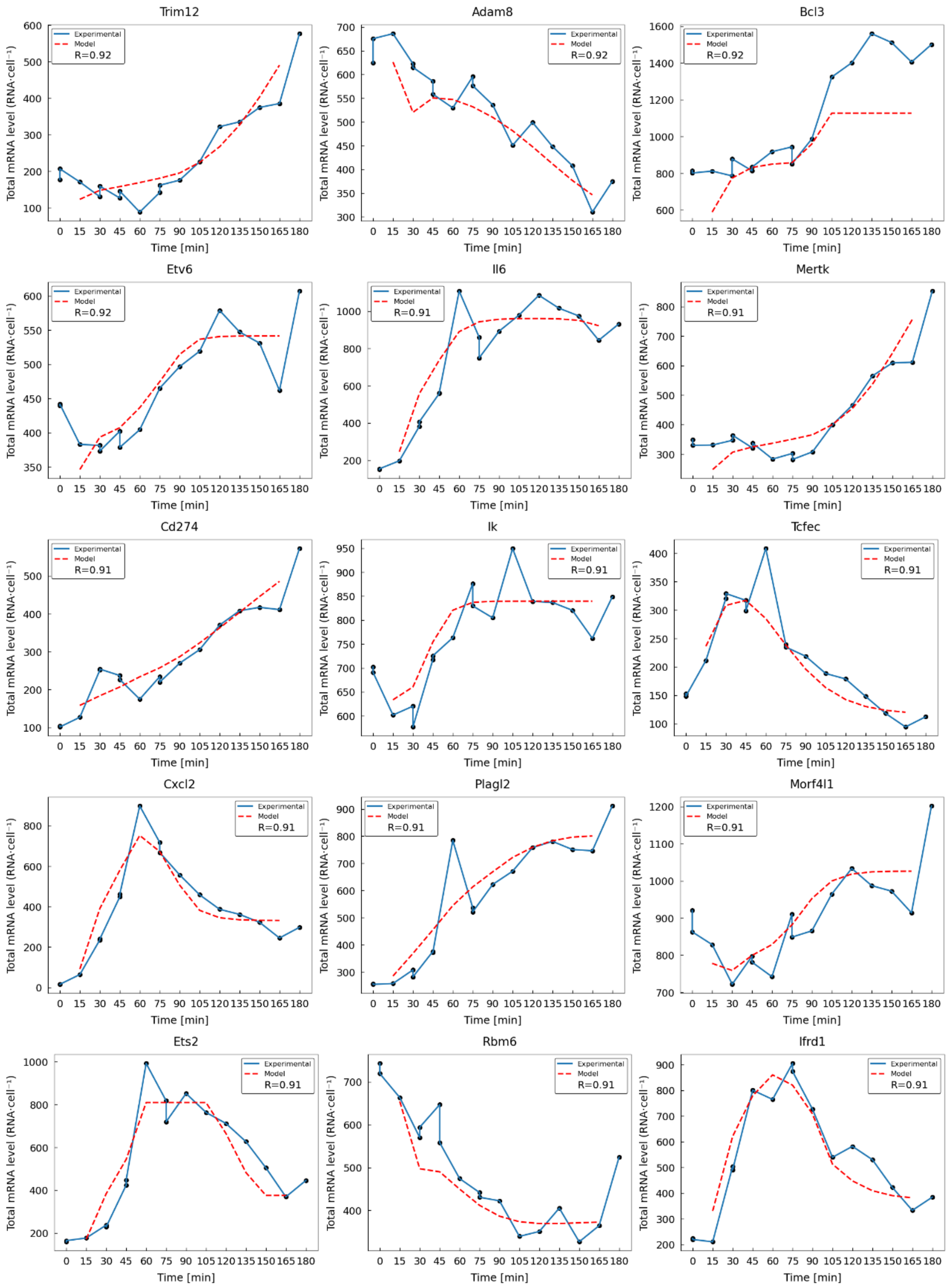

Figure S5 continued.

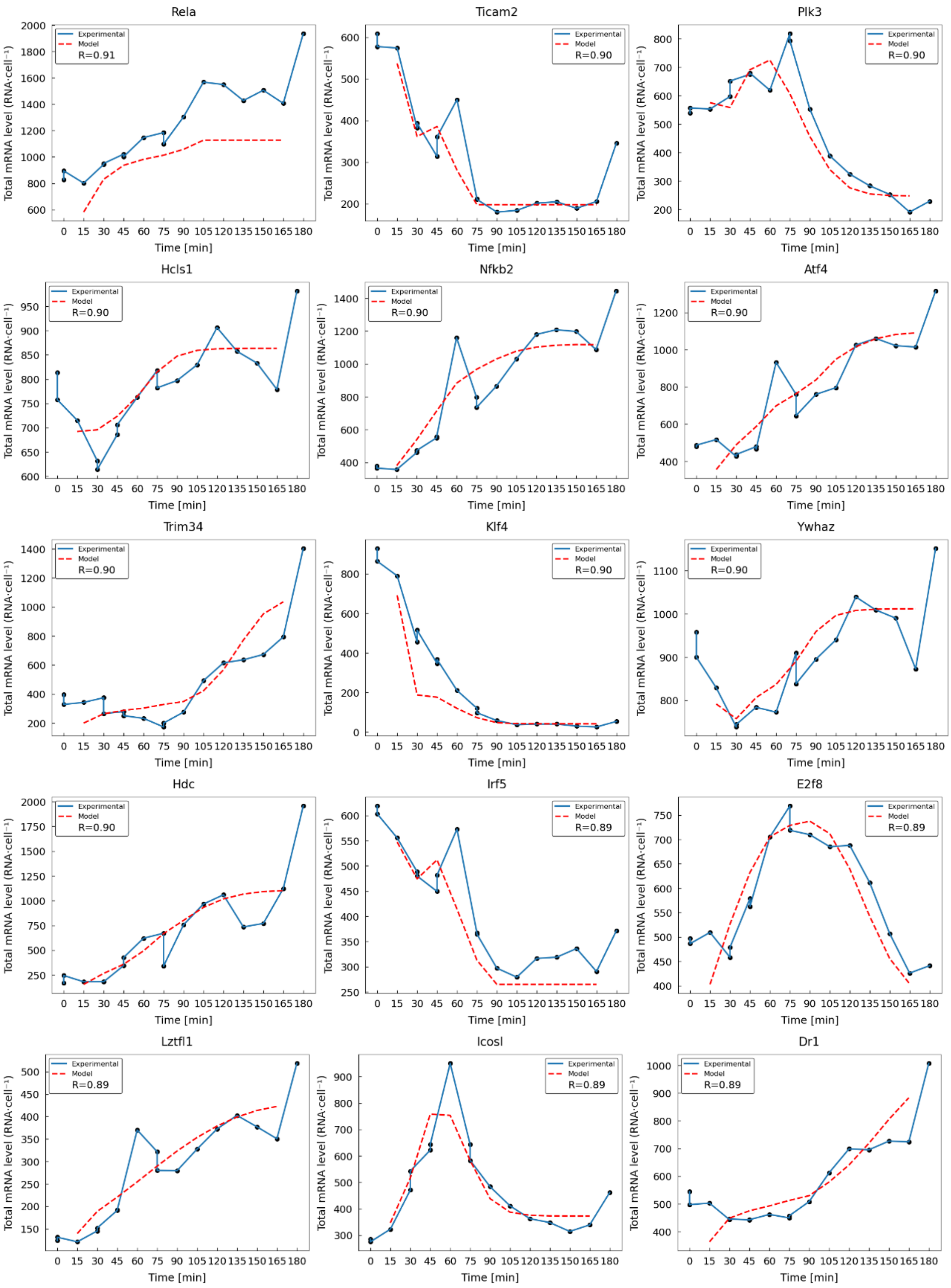

Figure S5 continued.

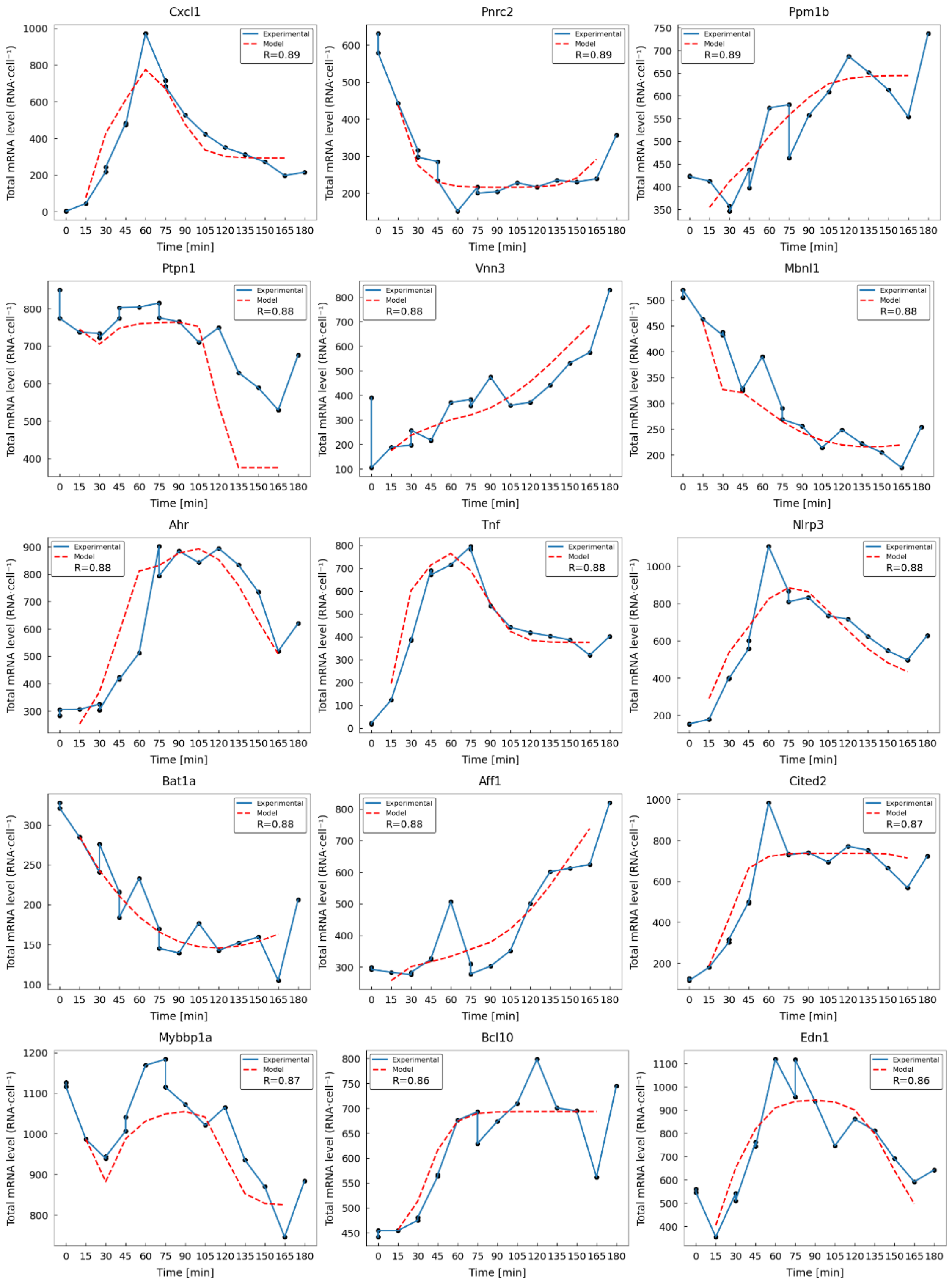

Figure S5 continued.

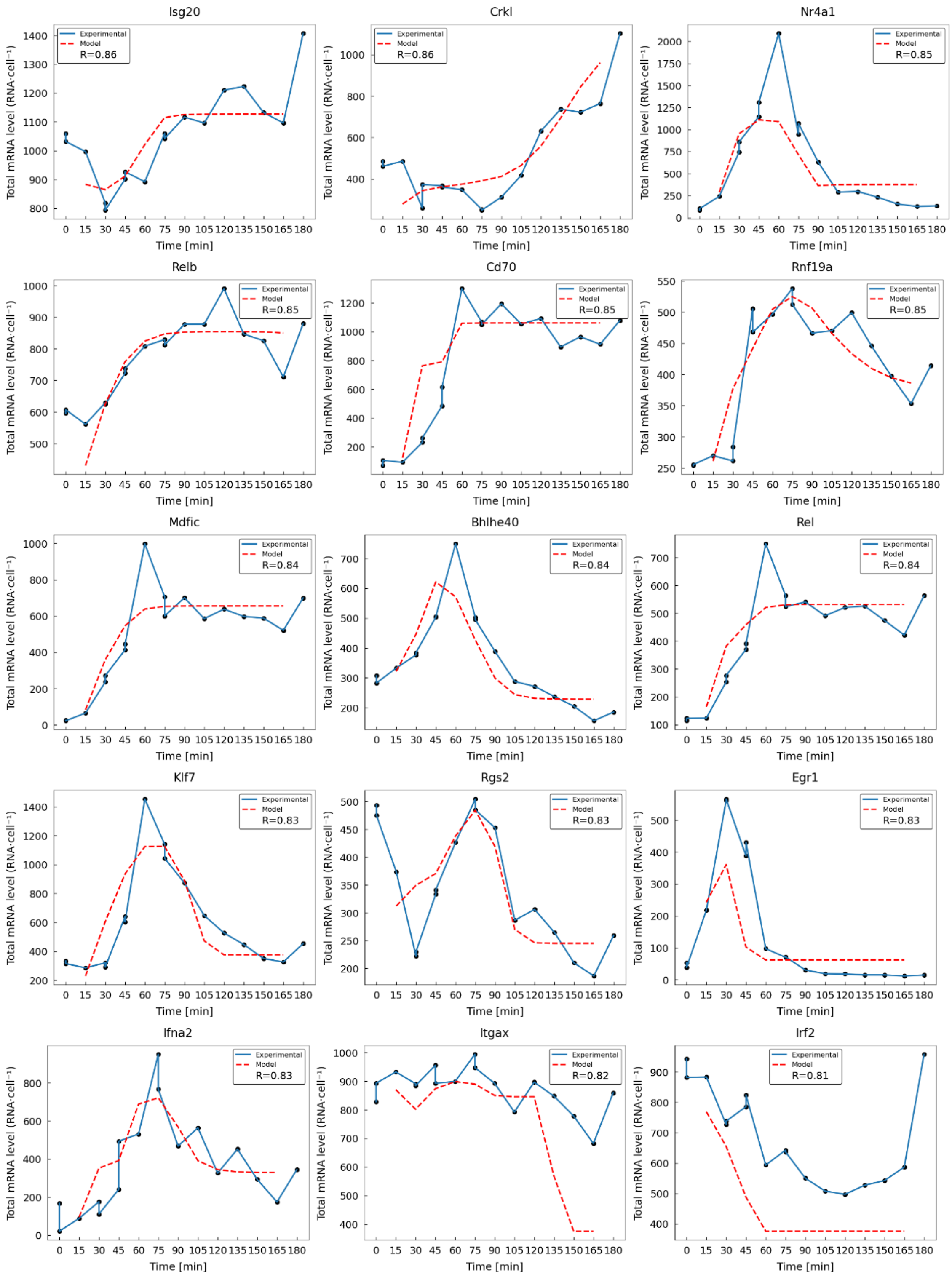

Figure S5 continued.

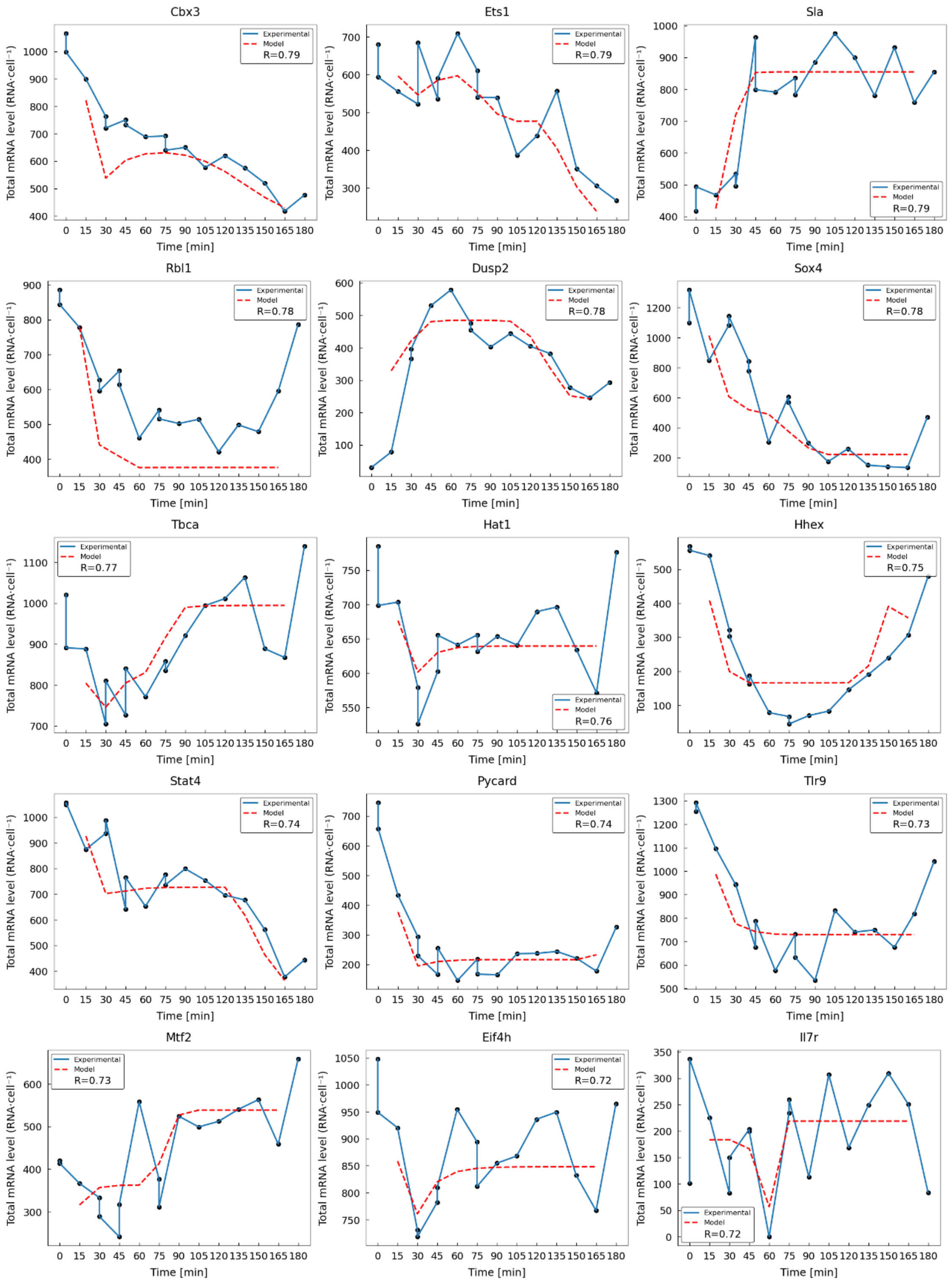

Figure S5 continued.

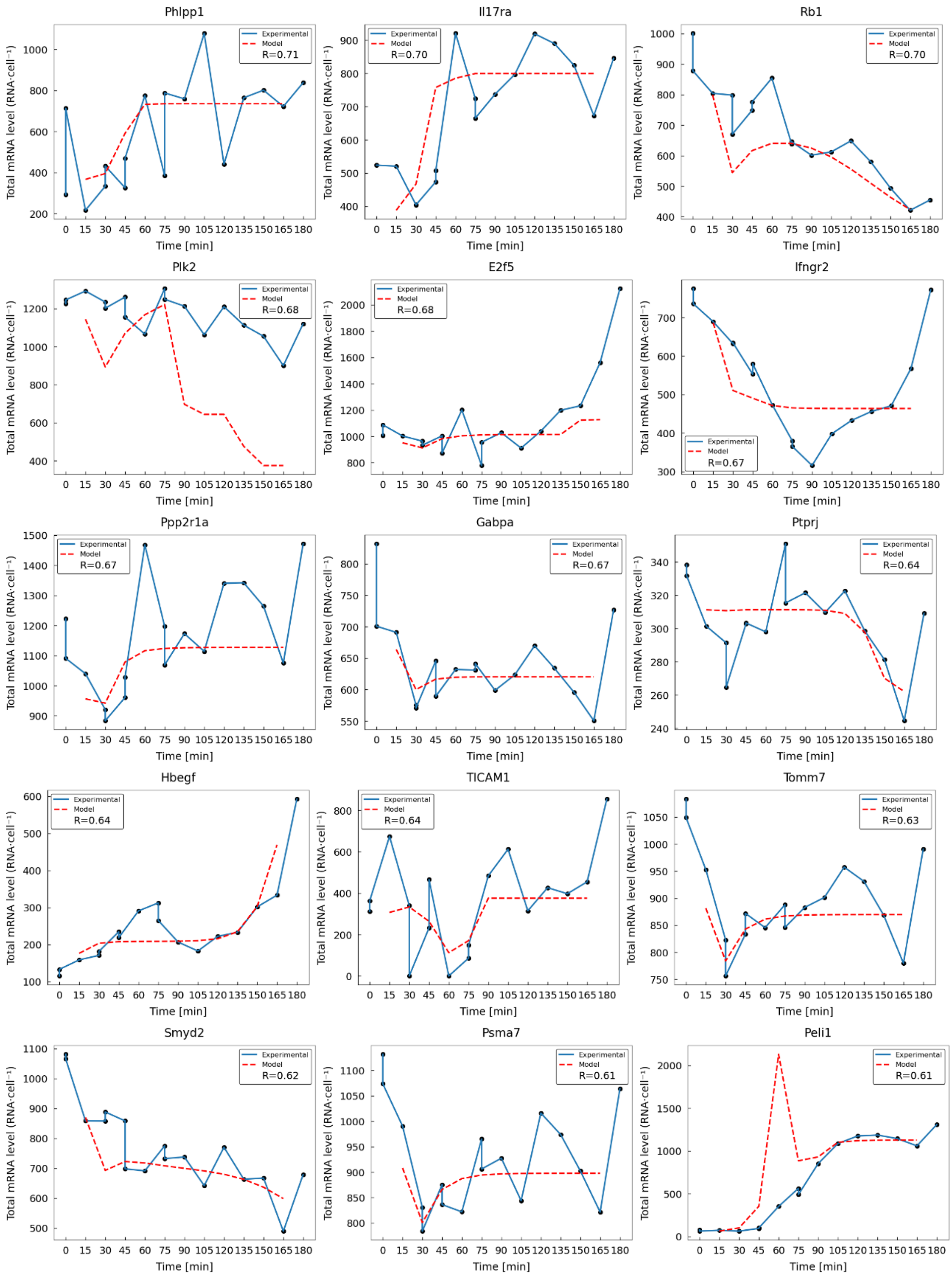

Figure S5 continued.

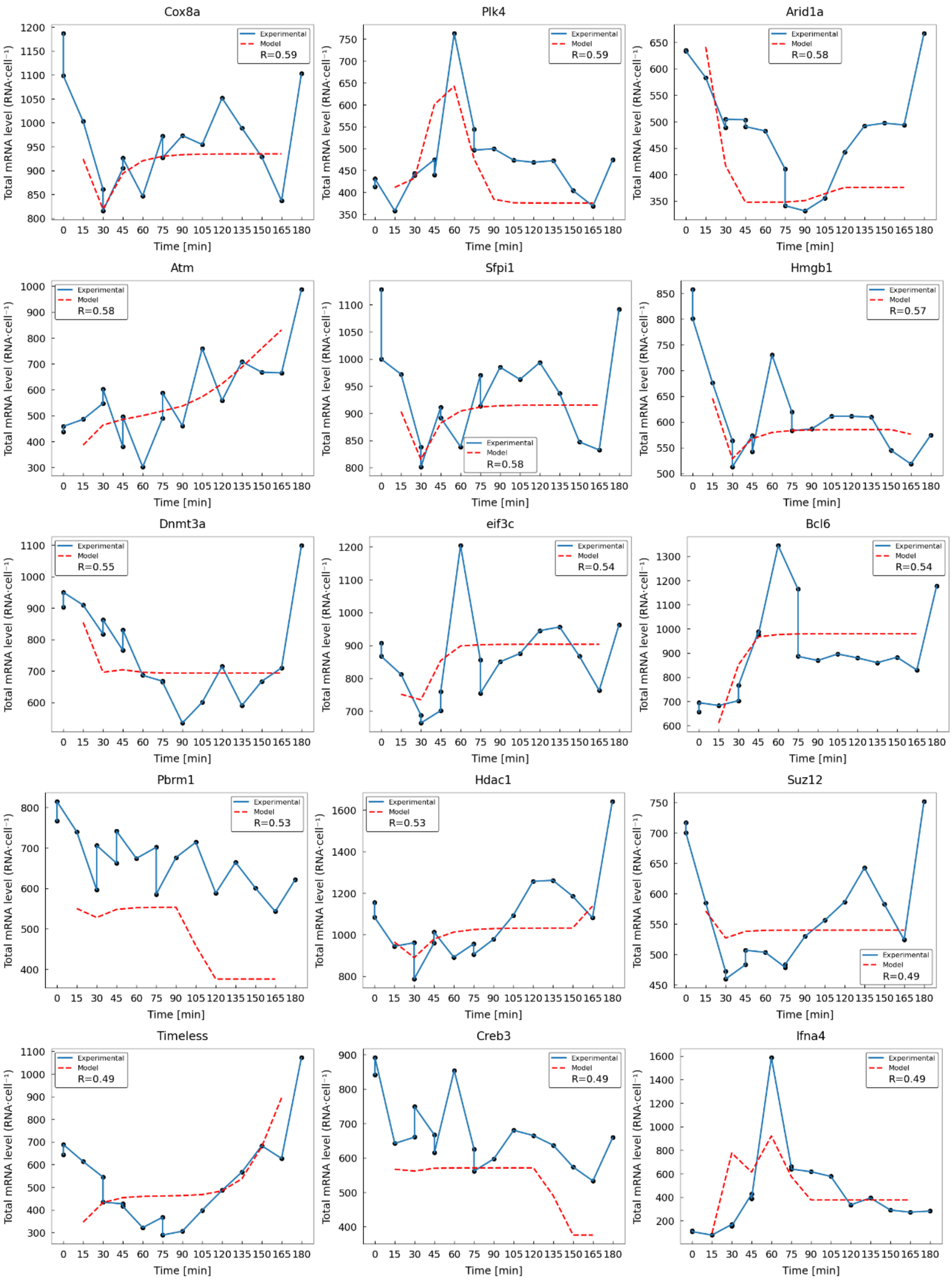

Figure S5 continued.

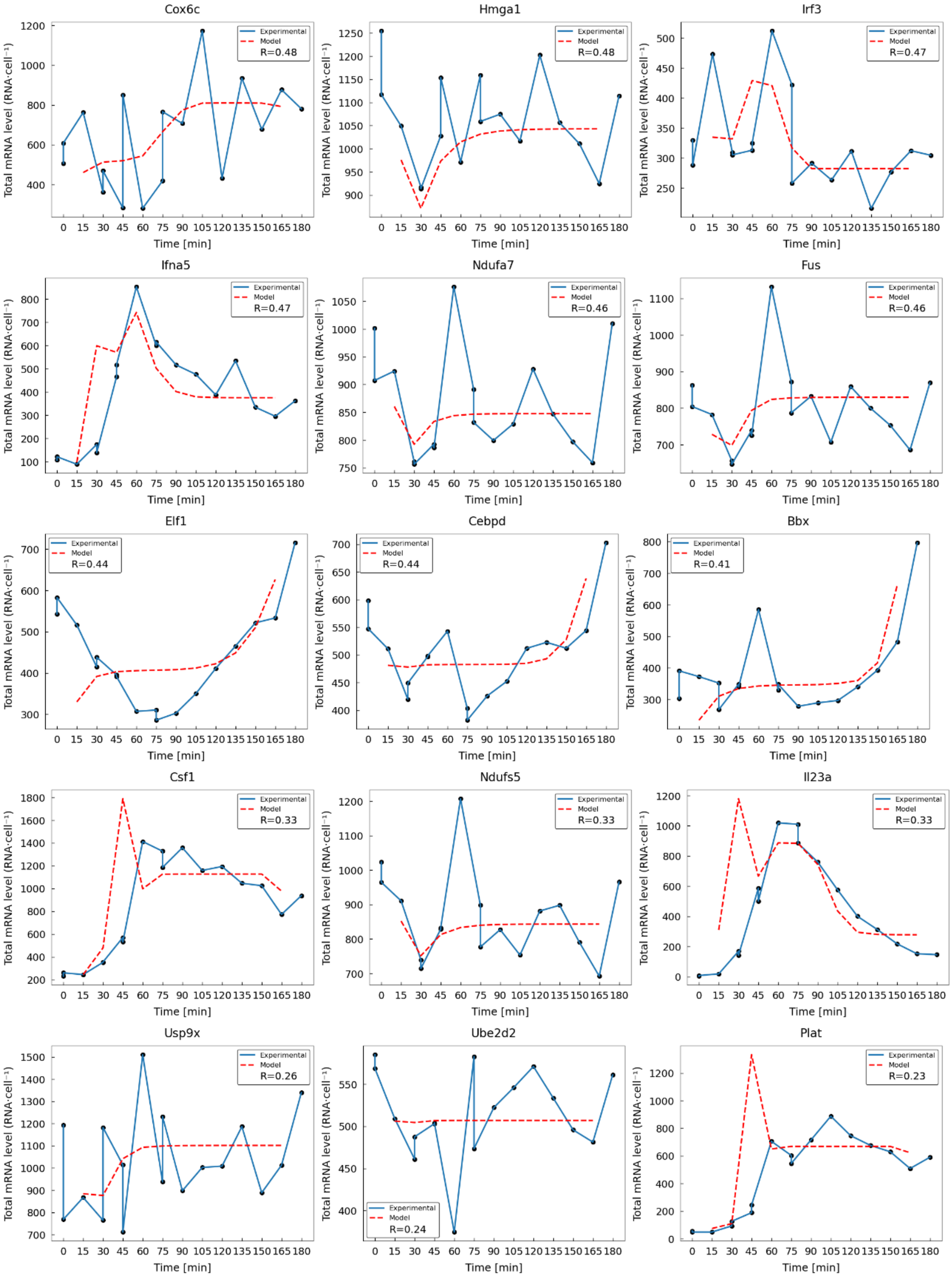

Figure S5 continued.

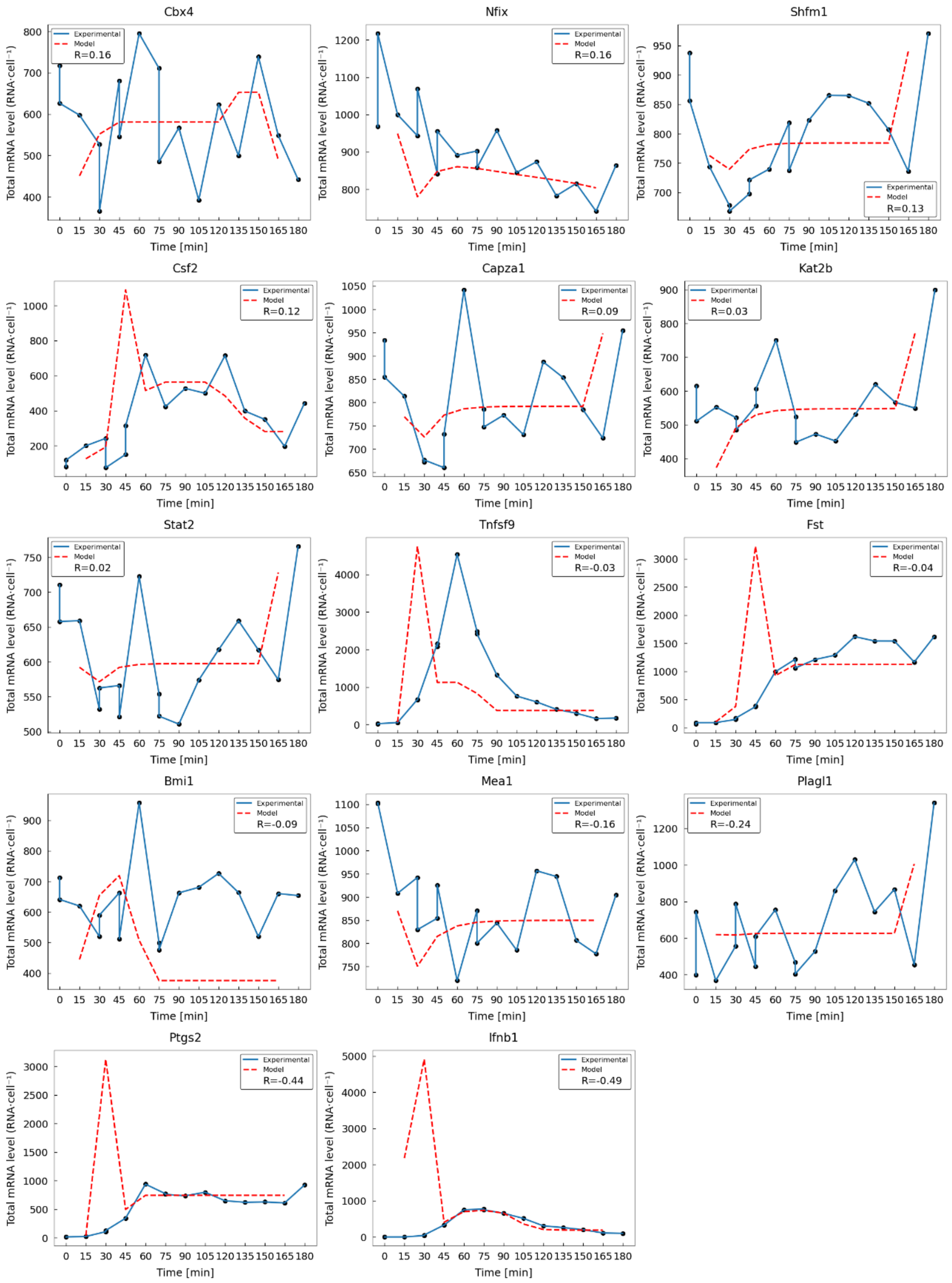

Figure S5 continued.

4 References

1. Rabani, M., Levin, J., Fan, L. *et al.* Metabolic labeling of RNA uncovers principles of RNA production and degradation dynamics in mammalian cells. *Nat Biotechnol* **29**, 436–442 (2011). doi: 10.1038/nbt.1861

2. Chechik, G. & Koller, D. Timing of gene expression responses to environmental changes. *J Comput Biol* **16**, 279-290 (2009). doi: 10.1089/cmb.2008.13TT

3. Dass, C. R., Saravolac, E. G., Li, Y., Sun, L. Cellular Uptake, Distribution, and Stability of 10-23 Deoxyribozymes. *Antisense Nucleic Acid Drug Dev* **12**, 289–299 (2002). doi: 10.1089/108729002761381276

4. Tang, F., Lao, K., Surani, M. A. Development and applications of single-cell transcriptome analysis. *Nat Methods* **8**, 6–11 (2011). doi: 10.1038/nmeth.1557

5. Kurimoto, K., Yabuta, Y., Ohinata Y., *et al.* An improved single-cell cDNA amplification method for efficient high-density oligonucleotide microarray analysis, *Nucleic Acids Res* **34**, e42(2006). doi: 10.1093/nar/gkl050

6. Krebs, J. E., Kilpatrick, S. T., Lewin, B., Goldstein, E. S. (2014). Lewin's Genes XI, p135. Japan: Jones & Bartlett Learning.

7. Shapiro, E., Biezuner, T., & Linnarsson, S. Single-cell sequencing-based technologies will revolutionize whole-organism science. *Nat Rev Genet* **14**, 618–630 (2013). doi: 10.1038/nrg3542

8. Kawasaki E. S. Microarrays and the gene expression profile of a single cell. *Ann N Y Acad Sci* **1020**, 92–100 (2004). doi: 10.1196/annals.1310.010

9. Marinov G. K., Williams B. A., McCue K., *et al.* From single-cell to cell-pool transcriptomes: stochasticity in gene expression and RNA splicing. *Genome Res* **24**, 496-510 (2014). doi: 10.1101/gr.161034.113.

10. Borggräfe, J., Victor, J., Rosenbach, H., *et al.*, Time-resolved structural analysis of an RNA-cleaving DNA catalyst. *Nature* **601**, 144-149 (2022). doi: 10.1038/s41586-021-04225-4.

11. Schubert, S., Gül, D. C., Grunert, H., *et al.*, RNA cleaving '10-23' DNAzymes with enhanced stability and activity, *Nucleic Acids Res* **31**, 5982–5992 (2003), doi: 10.1093/nar/gkg791

12. Brundel B., Van Gelder I., Henning R., *et al.* Alterations in potassium channel gene expression in atria of patients with persistent and paroxysmal atrial fibrillation: differential regulation of protein and mRNA levels for K+channels. *J Am Coll Cardiol* **37**, 926–932 (2001). doi: 10.1016/S0735-1097(00)01195-5

13. Perl K., Ushakov K., Pozniak Y. *et al.* Reduced changes in protein compared to mRNA levels across non-proliferating tissues. *BMC Genomics* **18**, 305 (2017). doi: 10.1186/s12864-017-3683-9

14. Liu, Y., Beyer, A., Aebersold, R. On the Dependency of Cellular Protein Levels on mRNA Abundance, *Cell* **165**, 535-550 (2016). doi: 10.1016/j.cell.2016.03.014

15. Gygi S. P., Rochon Y., Franza B. R., Aebersold R. Correlation between protein and mRNA abundance in yeast. *Mol Cell Biol* **19**, 1720-30 (1999). doi: 10.1128/MCB.19.3.1720.
